# Unique Sertoli cell adaptations support enhanced spermatogenesis in chickens

**DOI:** 10.1186/s40104-025-01304-8

**Published:** 2025-12-10

**Authors:** Gaoqing Xu, Zhuoxuan Gu, Ziming Wang, Jing Zhao, He Ding, Hongyu Liu, Yi Fang, Xin Ma, Jing Guo, Wenfa Lyu, Jun Wang

**Affiliations:** 1 College of Animal Science and Technology, Jilin Agricultural University, 2888 Xincheng Street, Changchun, Jilin Province 130118 China; 2 College of Animal Science, Anhui Science and Technology University, Chuzhou, 233100 China; 3 Key Laboratory of Animal Production, Product Quality and Security, Ministry of Education, Jilin Agricultural University, Changchun, Jilin Province, China

**Keywords:** Chicken testes, Leydig cells, Sertoli cells, Single-cell RNA sequencing, Spermatogenesis

## Abstract

**Background:**

The cellular basis of testicular development and spermatogenesis for the extreme sperm density in chickens (100-fold higher than mammals) remains poorly defined. A comprehensive understanding of the molecular characteristics driving poultry testicular development is crucial for explaining this enhanced spermatogenic capacity.

**Results:**

Here, we first established a single-cell transcriptome profile of chicken testes from hatching to maturity, identifying the dynamic transcriptional characteristics of germ cell fate transition and exploring the developmental characteristics of Sertoli cells and Leydig cells. Multi-species comparisons revealed a higher proportion of germ cells and the unique adaptations of Sertoli cells in chicken testes. Most importantly, our results demonstrated that Sertoli cells dominated in somatic composition of mature chicken testes, and proliferating Sertoli cells persisted in chicken testes even after sexual maturity, while no proliferating Sertoli cells in mammals. We also found a richer interaction network between chicken testicular cells, especially the specific activation of Sertoli cell interaction signals, such as TGF-β, BMP, EGF, and activin. These adaptations of Sertoli cells may support the spermatogenic superiority in chickens. Additionally, our results indicated that cAMP responsive element binding protein 5 (*CREB5*) played a crucial role in maintaining the maturation and function of chicken Sertoli cells, and circadian rhythm promoted testosterone secretion and the development of Leydig cells.

**Conclusion:**

Our study revealed that the sustained proliferative capacity of Sertoli cells, their enriched signaling network, and the regulatory roles of CREB5 and circadian rhythms collectively represented unique testicular adaptations in chickens. These findings may hold extraordinary significance in understanding the molecular characteristics of poultry testicular development, and provide a plausible framework for explaining enhanced spermatogenesis in poultry.

**Graphical Abstract:**

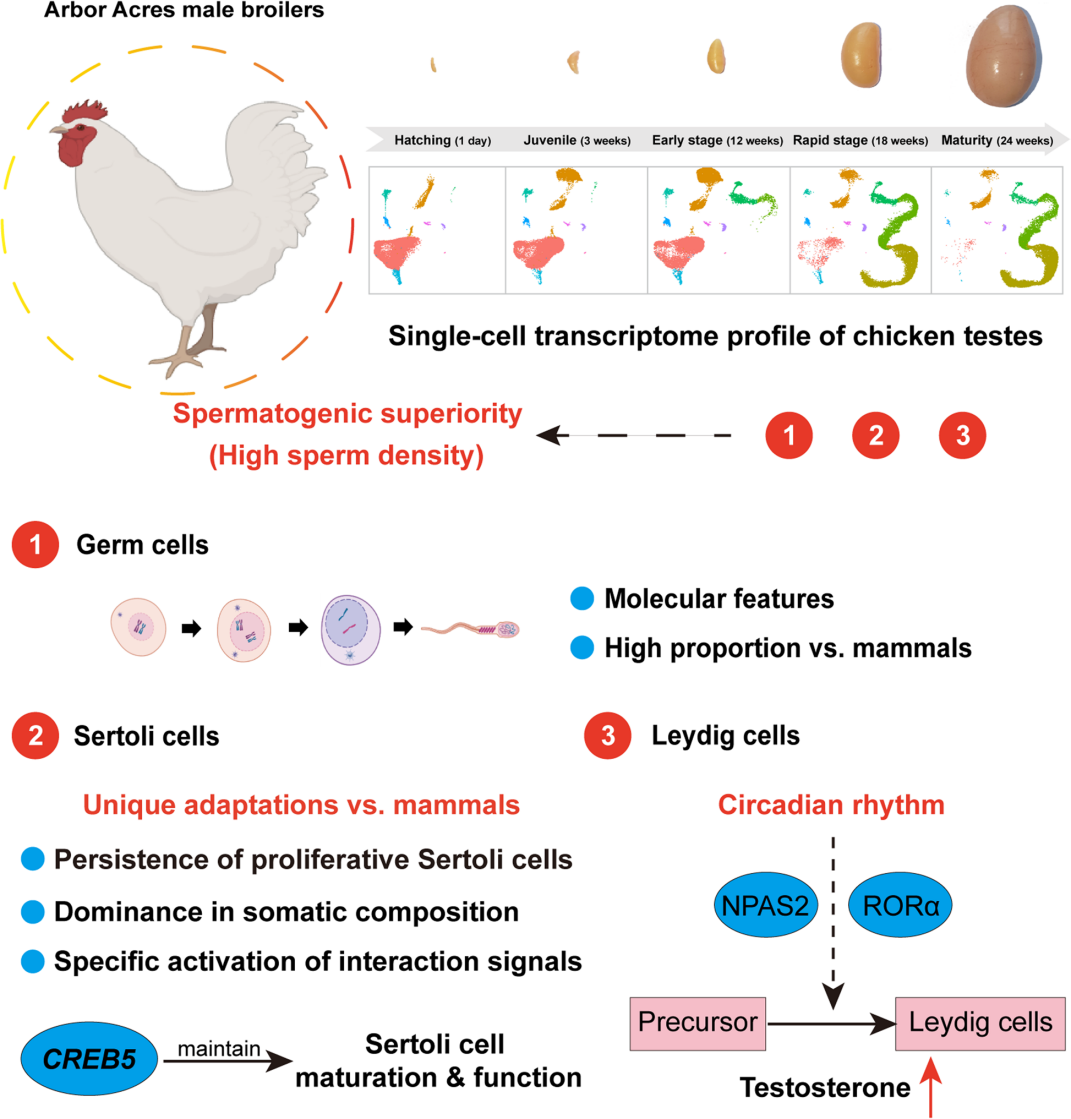

**Supplementary Information:**

The online version contains supplementary material available at 10.1186/s40104-025-01304-8.

## Introduction

The testis is a crucial reproductive organ in male animals, serving as the site for spermatogenesis, which generates sperm to transmit genetic information [1]. Spermatogenesis is a precise and dynamic developmental process that involves the differentiation of spermatogonial stem cells (SSCs), mitosis of spermatogonia, meiosis of spermatocytes, morphological changes in spermatids and the formation of sperm [2]. However, the higher sperm density were demonstrated in poultry (3 × 10^9^ mL^−1^ in chicken) than that in mammals, such as humans (2 × 10^7^ mL^−1^) and mice (1 × 10^7^ mL^−1^) [3–5]. Additionally, chickens, as light sensitive animals, have highly complex and diverse circadian rhythm systems, and heavily rely on their circadian rhythms to regulate reproductive processes [6, 7]. Prolonging the photoperiod has become an important means to promote testicular spermatogenesis during chicken production [8, 9]. These characteristics indicate the existence of unique adaptations during chicken spermatogenesis. However, the molecular basis of testicular development and spermatogenic superiority in poultry remains unclear.

During spermatogenesis, Sertoli cells and Leydig cells, and their secretions, especially testosterone secreted by Leydig cells, are important components of the testicular microenvironment that support germ cell development and sperm formation [10]. Sertoli cells, as the only somatic cells that directly interact with developing germ cells, can form the blood-testis barrier (BTB) and have been described as key drivers of spermatogenesis [11]. The number of Sertoli cells directly determines the testicular size and sperm production [12]. Immature Sertoli cells have strong phagocytic ability to promptly engulf underdeveloped or defective germ cells, and mature Sertoli cells provide protection and nutrition to germ cells [13]. In mammals, Sertoli cells exist in an immature state before puberty and transform into mature Sertoli cells that no longer differentiate during puberty [14]. However, there is limited understanding of the developmental characteristics of germ cells, Sertoli cells, and Leydig cells in chicken testes.

Here, we investigated chicken testes at five stages: the hatching stage (chicken_1d), juvenile stage (chicken_3w), developmental stage (chicken_12w), rapid development stage (chicken_18w), and mature stage (chicken_24w) using single-cell transcriptomics (10 × Genomics) to explore the molecular characteristics of spermatogenesis and spermatogenic superiority in chicken testes. We identified and characterized the immature and mature subtypes of Sertoli cells in chicken testes. Most importantly, we verified the unique persistence of proliferating immature Sertoli cells in chicken testes after sexual maturity through immunofluorescence, cell cycle detection and EDU staining. We preliminarily elucidated the effects of cAMP responsive element binding protein 5 (*CREB5*) on maintaining the maturation and function of chicken Sertoli cells. We also identified precursor cells of Leydig cells and detected the effects of circadian rhythm on testosterone secretion and the development of Leydig cells through retinoic acid receptor-related orphan receptor alpha (*RORα*) and neuronal PAS domain protein 2 (*NPAS2*) interference. Our findings not only provide substantial data on the molecular features of testicular development and spermatogenesis in poultry, but also lay the foundation for the developmental mechanisms of Sertoli cells and Leydig cells.

## Method details

### Testis sample preparation

Arbor Acres male broiler chickens (*Gallus gallus* domesticus) were randomly selected from a breeding farm with a standardized manner in Jilin Province, China. Chickens were housed in dry, well-ventilated coops with an activity space of 0.2 square meters per bird. A daily photoperiod of 12 h (combining natural and artificial light) is provided to stimulate normal physiological rhythms. The rearing environment is kept clean, quiet, and free from excessive stress. The testicular tissue samples were collected from the bilateral testes under sterile conditions. Specifically, the samples were obtained from the mid-region of the testes. Testis samples for scRNA-seq and immunostaining were obtained from 13 male Arbor Acres broilers of five different ages, namely, 1 d (*n* = 2), 3 weeks (*n* = 2), 12 weeks (*n* = 3), 18 weeks (*n* = 3), and 24 weeks (*n* = 3). Fresh testis samples were taken from the broilers and placed in phosphate-buffered saline (PBS). The left testes were selected and divided into two parts for single-cell sequencing and immunofluorescence. The experiments were approved by the Experimental Animal Welfare and Ethics Committee of Jilin Agricultural University (Approval No. 20210422 002) and performed in accordance with the protocol outlined in the “Guide for Care and Use of Laboratory Animals” (Jilin Agricultural University).

### Single-cell suspension preparation

Tissues were washed twice, decapsulated and minced into small pieces for adequate digestion. Suitable tissue pieces were then transferred to a 15-mL tube and digested through a standard two-step procedure. Briefly, tissues were sequentially digested with collagenase type IV (Cat# C5138-500MG, Sigma-Aldrich, St. Louis, MO, USA) and 0.25% trypsin/EDTA (Cat# 25300054, Invitrogen, Carlsbad, CA, USA). Subsequently, the digested cells were collected and subjected to a red blood cell (RBC) removal process using red blood cell (RBC) lysis buffer (CST, Cat# 46232). Single cells were obtained by filtering with 70 µm strainers (Thermo Fisher Scientific, Cat# 08-771-2) and collected into 1.5-mL tubes by centrifugation at 400 × *g* for 5 min. The cells were then detected by an automatic cell fluorescence analyzer (Count Star, Shanghai, China) and resuspended at approximately 400 cells/μL in 1 × PBS containing 0.4% BSA (Thermo Fisher Scientific, Cat# AM2616).

### scRNA-seq library preparation and sequencing

scRNA-seq was performed by CapitalBio Technology (Beijing, China) using the 10 × Genomics system. Briefly, after the cells were diluted, the cell suspension was loaded onto a 10 × Chromium Controller to generate single-cell gel beads in the emulsion using a Chromium Single Cell G Chip Kit (1000120, 10 × Genomics, Pleasanton, CA, USA) and a Gel Bead Kit V3.1 (1000121, 10 × Genomics, Pleasanton, CA, USA). In each experiment, approximately 10,000 single cells were captured from each testis sample. Captured cells were lysed to release RNA, and cDNA was generated by reverse transcription and amplified using an S1000 Thermal Cycler (Bio-Rad, USA). The cDNA quality was subsequently assessed on an Agilent 4200 TapeStation. Subsequently, scRNA-seq libraries were prepared using the Single Cell 3′ Library and Gel Bead Kit V3.1 following the manufacturer’s instructions. scRNA-seq libraries were sequenced at 150 bp paired ends (PE150) using an Illumina NovaSeq 6000 sequencer (Illumina, San Diego, CA, USA).

### Quality control and analysis of scRNA-seq data

We used Gallus_gallus GRCg6a.101 as the reference genome and then quantified the cell barcodes with CellRanger (https://support.10xgenomics.com/single-cell-gene-expression/software/downloads/latest). We imported the matrices into Seurat v4.0.4 (https://github.com/satijalab/seurat) and filtered low-quality cells as follows: cells expressing genes in fewer than three cells, cells with fewer than 800 or more than 7,000 genes, and cells with a percentage of mitochondrial genes greater than 5% were removed. Then, we used the “Merge” function to merge the scRNA-seq data of broiler testes from the same period. The merged data were normalized by “LogNormalize” and calculated for 2,000 highly variable genes by “FindVariableFeatures”. Next, we used “FindIntegrationAnchors” and “IntegrateData” to integrate the scRNA-seq data from the five periods, and the integrated data were normalized to “ScaleData”. We then performed principal component analysis (PCA) via “RunPCA” and selected 15 principal components (PCs). After that, we used the “FindNeighbors” and “FindClusters” functions to cluster the cells and visualized the data with the “RunUMAP” function.

### Integration analysis of scRNA-seq data

We used the default Wilcoxon rank-sum test in the “FindAllMarkers” function to determine the differentially expressed genes (DEGs) of each cell cluster with a log_2_ foldchange > 0.25. According to the DEGs of each cell cluster, we identified the cell types of each cluster by using well-known marker genes and integrated the same cell type. Subsequently, we extracted testicular germ cells through the "subset" function to perform germ cell clustering analysis again and identified the cell types of the cell clusters. Additionally, DEGs between different cell types or different periods of the same cell type were obtained through the "FindMarker" function with the parameters “logfc. threshold = 0.25”, “min. pct = 0.25”, and "p_val < 0.05". Moreover, Gene Ontology (GO) and Kyoto Encyclopedia of Genes and Genomes (KEGG) analyses of DEGs were performed using DAVID (https://david.ncifcrf.gov/) with *P* < 0.05 as the cutoff for significantly enriched terms and then visualized by the ggplot2 package (v3.3.5, https://r-graph-gallery.com/ggplot2-package.html).

### Cell trajectory analysis

The Monocle 2 package (v2.18.0, http://cole-trapnell-lab.github.io/monocle-release/docs/) was installed in R to reconstruct the cell differentiation trajectory. We identified DEGs that varied in pseudotime order and set “qval < 0.01” and “num_cells_expressed > 10” for pseudotime analysis. Next, we applied the "DDRTree" method of the "reduceDimension" function for dimensionality reduction and used the "orderCells" function to sort cells and construct trajectories. Cell visual trajectories of clusters and groups were generated in pseudotime order using the "plot_cell_trajectory" function and colored by pseudotime values, cell states, and original ident. Pseudotime heatmaps were generated using the "plot_pseudotime_heatmap" function, and pseudotime genes were visualized using the “plot_genes.in_pseudotime” function.

Additionally, RNA Velocity analysis was used to detect the development of Leydig cells. The velocyto package (https://velocyto.org/) and scVelo (https://scvelo.readthedocs.io/en/stable/) package was installed in R to draw the cell differentiation trajectory under the uniform manifold approximation and projection (UMAP) clustering results.

### Cell‒cell communication analysis

The CellChat package (1.5.0) was installed in R to study the interactions between different cell types in testes [15]. Subsequently, we used the "computeCommunProb" and "aggregateNet" functions to compute communication probabilities and infer cellular communication networks and used the "compareInteractions" function to compare the number and strength of interactions. Subsequently, we visualized the overall signaling patterns between humans and chickens by using the “netAnalysis_signalingRole_heatmap” function.

### Cross-species correlation analysis

To compare and observe the differences in cell composition and gene expression in testicles of different species, we reintegrated single-cell transcriptome data from 24-week-old broilers (*n* = 3) and collected data from adult humans (*n* = 5) and adult mice (*n* = 2) to perform cross-species correlation analysis. We eliminated batch and technical differences through a unified integration approach. The single-cell transcriptome data of both adults and mice were obtained from ArrayExpress with the accession codes E-MTAB-11063 (human) and E-MTAB-11071 (mouse) [16].

### Histological analysis

The collected fresh testis tissues were soaked in a 4% paraformaldehyde (PFA) solution and dehydrated using gradient ethanol (30%, 50%, 70%, 95%, and 100%, 2 h of each gradient). The dehydrated tissues were placed in xylene for 20 min and embedded in paraffin. Next, the embedded testis tissues were cut into 5 μm sections by a microtome (Leica RM2235, Wetzlar, Germany) and then successively placed in xylene for deparaffinization and gradient alcohol (100%, 95%, 75%, 50%, and 30%) for 5 min each for rehydration. After these steps, hematoxylin and eosin (H&E) was used to stain the sections for a few seconds. Subsequently, the slices were rinsed with water to remove excess dye, sealed with neutral balsam (Biotopped, Beijing, China), and finally analyzed using a Leica microscope (Wetzlar, Germany).

### Immunofluorescence of testes

Testis tissues of chicken from different groups and adult mouse were soaked in a 4% PFA solution, dehydrated, and then embedded in paraffin. Next, the embedded testis tissues were cut into 5 μm sections. The sections were successively placed in xylene and gradient alcohol for 5 min each and treated with an 80 °C sodium citrate solution for 20 min. After being washed with PBS, the sections were blocked with 10% sheep serum for 30 min, followed by incubation with a primary antibody overnight at 4 °C, including ACTA2 (Invitrogen, Cat# MA5-41117), CLEC3B (Invitrogen, Cat# MA5-34760), VWF (Proteintech, Cat# 66682-1-lg), DDX4 (Invitrogen, Cat# MA5-15565), TOP2A (Invitrogen, Cat# MA5-32096), TPPP2 (Invitrogen, Cat# PA5-112776), SOX9 (Millipore, Cat# AB5535), and PCNA (Proteintech, Cat# 10205-2-AP). After that, the sections were incubated with secondary antibody for 1 h at room temperature, followed by incubation with DAPI as a nuclear stain. Finally, the sections were detected, and images were collected using a fluorescence microscope.

### Extraction of Leydig cells and Sertoli cells

Leydig cells and Sertoli cells were isolated from the testicular tissues of 24-week-old broiler chickens via previously described methods [6, 17, 18]. The cells were cultured in DMEM/F12 (Gibco, Waltham, Massachusetts, USA) supplemented with 10% fetal bovine serum (Sangon Biotech, Shanghai, China) and placed in a 37 °C incubator with 5% CO_2_. When the cell purity exceeded 95%, the cells were used for subsequent experiments.

### Cell proliferation detection

Sertoli cell proliferation was detected using a BeyoClick™ EdU-594 cell proliferation kit (Beyotime Institute of Biotechnology, Shanghai, China). Sertoli cells were cultured in triplicate in 24-well plates at a density of 5 × 10^4^ cells/well and treated following the manufacturer’s instructions. Finally, the EdU-positive cells were detected by a cell imaging detector (BioTek, Winooski, VT, USA).

### Cell cycle detection

The purified chicken Sertoli cells were collected by centrifugation at 500 × *g* for 5 min. The cells were then washed once with PBS, centrifuged again, and the supernatant was discarded. The 500 μL PBS containing 50 μg/mL PI staining solution (Beyotime Institute of Biotechnology, Shanghai, China) and 100 μg/mL RNase A were add in cells. After gentle mixing, the cells were incubated in the dark for 30 min at 37 °C. Data were collected using a flow cytometer (ACEA Biosciences, CA, USA), with appropriate gating conditions set to exclude debris and doublets. The proportions of cells in the G1, S, and G2/M phases of the cell cycle were analyzed based on PI fluorescence intensity.

### Gene interference

First, we designed and synthesized small interfering RNAs (siRNAs), including siCREB5, siRORα, siNPAS2 and NC (GenePharma, Suzhou, China). The sequences of the siRNAs used are shown in Table S1. Second, Leydig cells were seeded onto six-well plates at a density of 1 × 10^6^ cells/well and cultured overnight. When the density of adherent cells reached 70%, we transfected siCREB5 (50 nmol/L) into chicken Sertoli cells, and siRORα or siNPAS2 (50 nmol/L) into Leydig cells by using Lipofectamine 2000 following the manufacturer’s instructions (Sigma-Aldrich, St. Louis, MO, USA). After 6 h, 10% fetal bovine serum was added to all the wells, and the cells were cultured for 24 h.

### RT-qPCR analysis

To obtain total RNA, broiler Leydig cells were lysed by using TRIzol reagent (Invitrogen, Carlsbad, CA, USA). Next, 1 μg of RNA was reverse transcribed to cDNA using a reverse transcription kit (Takara, Tokyo, Japan). Subsequently, gene expression was detected by RT-qPCR using SYBR Premix reagent (Takara, Tokyo, Japan) and analyzed by the 2^−∆∆Ct^ method. β-Actin was used as a reference gene to calculate relative mRNA expression levels. The primer sequences of the detected genes and their annealing temperatures (Tm) are shown in Table S2.

### Testosterone measurement

After *NPAS2* interference, testosterone levels in Leydig cells were measured using an ELISA kit following the manufacturer’s instructions (J&L Biological, Shanghai, China). Ten microliters of supernatant, 40 µL of sample diluent and HRP-conjugate reagent were added sequentially to each well. After incubation at 37 °C for 1 h, testosterone levels were measured at 450 nm using a microplate reader (BioTek, Winooski, VT, USA).

### Western blot

Total protein was extracted using RIPA lysis buffer and measured using a protein assay kit (Beyotime, Shanghai, China). Equal amounts of protein (20 µg) were separated by 10% SDS–polyacrylamide gel electrophoresis (SDS-PAGE) and subsequently transferred onto polyvinylidene fluoride (PVDF) membranes. After blocked with 5% non-fat milk for 1 h, the membranes were incubated overnight at 4 °C with primary antibodies, including CREB5 (Proteintech, Cat# 14196-1-AP), NPAS2 (Santa cruz, Cat# sc-134404), RORα (Proteintech, Cat# 10616-1-AP), ZO-1 (Proteintech, Cat# 21773-1-AP), occludin (Proteintech, Cat# 27260-1-AP), STAR (OmnimAbs, Cat# OM153513), HSD3B1 (abcam, Cat# ab65156), CYP11A1 (CellSignalingTechnology, Cat# 14217). Then, the membranes were probed with appropriate horseradish peroxidase (HRP)-conjugated secondary antibodies, including goat anti-mouse IgG (Proteintech, Cat# SA00001-1) and goat anti-rabbit IgG (Proteintech, Cat# SA00001-2). Protein bands were visualized using an enhanced chemiluminescence (ECL) detection reagent and captured by a chemiluminescence imaging system. β-Actin (Proteintech, Cat# 60008-1-lg) was used as a loading control to ensure equal protein loading.

### Statistical analysis

Each experiment was conducted with 3 biological replicates, and all the data were statistically analyzed with GraphPad Prism version 5.0 (GraphPad Prism Software, San Diego, CA, USA). Differences between groups were determined by one-way analysis of variance (ANOVA) and Student’s *t*-test. A *P*-value < 0.05 indicated a statistically significant difference.

## Results

### Single-cell transcriptome of chicken testes from hatching to maturity

**Fig. 1:**
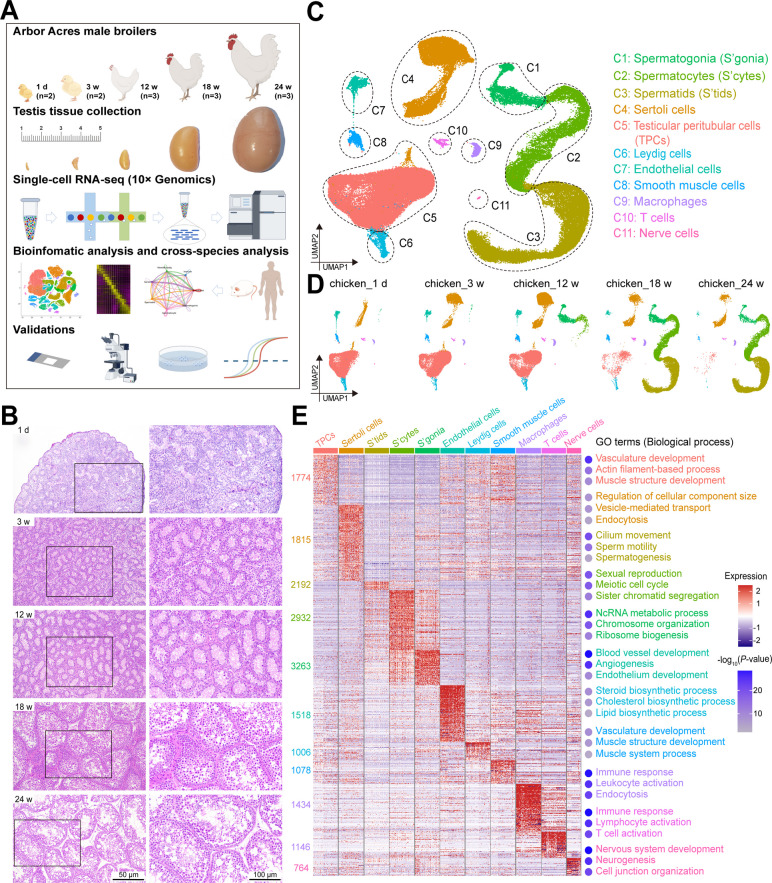
Single-cell transcriptome profile of chicken testes from hatching to sexual maturity. **A** Schematic of the experimental workflow. **B** Hematoxylin-eosin (HE) staining of section of chicken testes from five different ages (1 d, 3 weeks, 12 weeks, 18 weeks, and 24 weeks), demonstrating morphological changes of chicken testes during the development process from hatching to sexual maturity. **C** A UMAP plot showing the annotated testicular cell types. Each dot represents a single testicular cell and is colored based on its cell type. **D** Five UMAP plots showing the annotated testicular cell types from 1 d (*n* = 2), 3 weeks (*n* = 2), 12 weeks (*n* = 3), 18 weeks (*n* = 3), and 24 weeks (*n* = 3) chickens. **E** Heatmap showing the markers of each cell cluster. The number on the left shows the number of markers, and is colored based on its cell type (Table S1). The gene expression levels are colored based on Z score. The figure on the right shows 5 representative GO terms enriched in the marker genes of each cluster with −log _10_ (*P*-value) of each GO term colored based on the right color key

To investigate chicken testis development, we collected 13 pairs of whole chicken testes from five different ages: 1 d, 3 weeks, 12 weeks, 18 weeks, and 24 weeks, and observed a significant increase in the size and weight of chicken testicles during the five stages from hatching to maturity (Fig. 1A and Fig. S1B). Histological results showed the formation of seminiferous tubules from 1 d to 3 weeks, spermatogonia proliferation from 3 to 12 weeks, and complete spermatogenesis at 18 weeks and 24 weeks (Fig. 1B). These results suggested the comprehensive histological characterization and foundational insights of chicken testicular development phases.

We then performed scRNA-seq on all 13 testicular samples (*n* = 2 or 3) from broiler chickens using the 10 × Genomics Chromium platform. A total of 105,486 cells from the 155,664 collected cells were used for the further analysis after quality control (Fig. S1A and Fig. S1B). To characterize the cell types of chicken testes, the UMAP analysis was performed on scRNA-seq data (Fig. 1C and D; Fig. S1C and S1D). We identified 11 major types of testicular cells using known specific marker genes of each cell type in testes [16, 19]. The somatic compartment comprised Sertoli cells (marked by *ALDH1A1*^+^, *GSTA2*^+^, *SOX9*^+^), Leydig cells (*CYP17A1*^+^, *CYP11A1*^+^, *STAR*^+^), testicular peritubular cells (TPCs; *ACTA2*^+^, *DCN*^+^, *COL1A1*^+^), smooth muscle cells (*RGS4*^+^, *RGS5*^+^, *HTRA1*^+^), endothelial cells (*VWF*^+^, *CDH5*^+^, *SOX18*^+^), nerve cells (*CDH19*^+^, *NRN1*^+^, *ADGRB3*^+^), and immune cells, including macrophages (*AVD*^+^, *CD83*^+^, *C1QA*^+^), T cells (*CD3E*^+^, *ICOS*^+^, *CD28*^+^). The germinal lineage was categorized into spermatogonia (*FGFR3*^+^, *HMGB3*^+^, *DAZL*^+^), spermatocytes (*TOP2A*^+^, *SMC2*^+^, *NUSAP1*^+^), and spermatids (*TPPP2*^+^, *SPATA4*^+^, *TUBA8B*^+^) (Fig. 1C and Fig. S2A). We also identified markers for major cell types through immunofluorescence staining, including ACTA2, VWF, CLEC3B, SOX9, DDX4, TOP2A, and TPPP2 (Fig. S1E). Additionally, a total of 18,294 genes were explored for each cell type, which could serve as potential markers for chicken testicular cells (Table S3). The definition and the physiological functional characteristics of each cell type was further confirmed by Gene Ontology (GO) analysis (Fig. 1E).

Subsequently, we calculated the percentage composition of various testicular cell types at the five stages. The proportion of TPCs in testicular cells was highest at 1 d (81.10%) and 3 weeks (76.60%), while the proportion of Sertoli cells in testicular cells was highest at 12 weeks (40.29%), and Sertoli cells still had the highest proportion in somatic cells at 18 weeks and 24 weeks. Notably, the proportion of germ cells rapidly increased from 12 weeks (11.08%) to 18 weeks (78.54%), and reached as high as 91.19% in the 24 weeks (Fig. S2B). These results suggested that the development of germ cells and Sertoli cells may be related to the spermatogenic superiority in chickens.

### Dynamic transcriptional characteristics of germ cells during chicken spermatogenesis

**Fig. 2:**
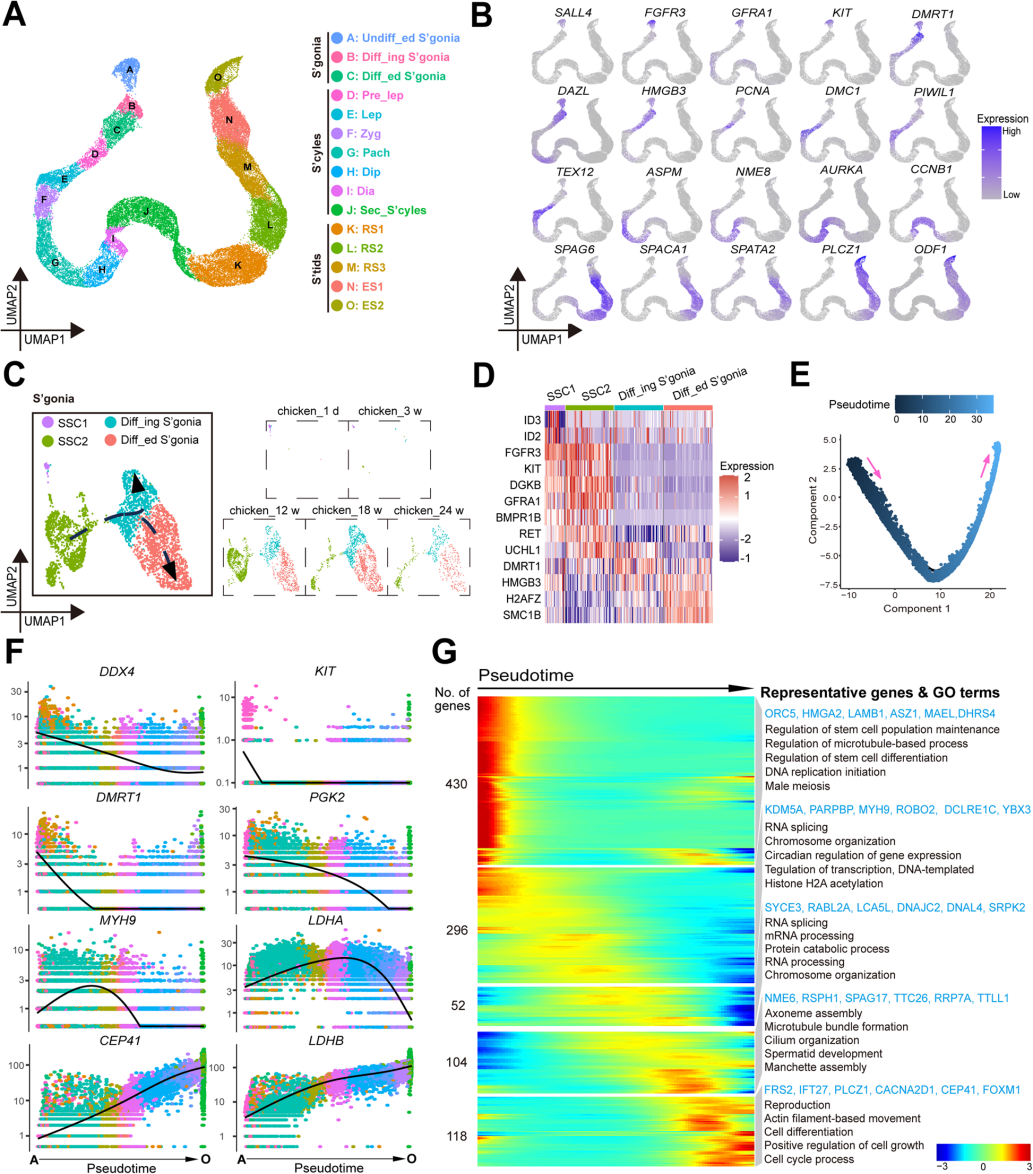
Dynamic transcriptome of germ cell in chicken testes at the single-cell resolution. **A** A UMAP plot showing the annotated cell types of testicular germ cells. Each dot represents a single germ cell and is colored based on its cell type. **B** Marker expression patterns of each cluster of testicular germ cells on UMAP plots. A gradient of blue and gray represents high or low marker expression levels. **C** UMAP plots showing the annotated cell types of spermatogonia. Dashed lines and arrows represent their developmental trajectory. **D** Heatmaps showing the representative markers of each cell cluster of spermatogonia. A gradient of red and blue represents high or low marker expression levels. **E** Pseudotime trajectory of testicular germ cells analyzed by Monocle analysis. Each dot represents a single germ cell and is colored based on the predicted pseudotime. **F** Expression patterns of representative dynamic genes as predicted by pseudotime during spermatogenesis. Germ cells are colored based on the cluster colors and ordered according to the pseudotime trajectory. Pseudotime (SSCs to ES2) is indicated below each gene plot column. **G** Heatmap showing the clusters of top 1,000 genes (Table S8) in pseudotime from testicular germ cells of broiler chickens. A gradient of red and blue represents high or low gene expression levels based on the expression color code. The number on the left shows the number of differentially expressed genes, and the right side shows the representative genes and GO terms

Elucidating the different stages and characteristics of germ cell development is crucial for understanding the process of spermatogenesis [19]. Therefore, we integrated and analyzed 42,561 chicken germ cells and identified 15 main cell clusters. We defined 15 cell clusters as undifferentiated spermatogonia (Undiff_ed S’gonia), Diff_ing S’gonia, and Diff_ed S’gonia in spermatogonia; defined spermatocytes as the preleptotene stage (pre_Lep), leptotene stage (Lep), zygotene stage (Zyg), pachytene stage (Pach), diplotene stage (Dip), diakinesis (Dia), and secondary spermatocytes (Sec_S’cyles) based on the stage of meiosis; and defined spermatids as round spermatids (RS) and elongated spermatids (ES) (Fig. 2A). We detected specific expression of *FGFR3* in SSCs; high expression of *DMRT1* in Diff_ing S’gonia; high expression of *PCNA* in pre_Lep; specific expression of *DMC1*, *PIWIL1*, *TEX12*, *ASPM*, and *NME8* in meiosis; high expression of *CCNB1* in Sec_S’cyles; high expression of *SPAG6* and *SPATA2* in RS; and high expression of *PLCZ1* in ES (Fig. 2B). According to the statistical analysis of the number and proportion of germ cells, only a small number of germ cells were present in chicken_1d and chicken_3w, and complete spermatogenesis occurred in chicken_18w and chicken_24w. Notably, the proportion of spermatids reached as high as 71.6% in chicken_24w (Fig. S3A).

Our results revealed 35,798 potential marker genes, most of which were dynamically transcribed and expressed during spermatogenesis (Fig. S3B; Table S4). We analyzed the expression patterns of key markers in each cluster during spermatogenesis. Notably, we identify two types of SSCs in Undiff_ed S’gonia by a detailed analysis of the subtypes, named SSC1 and SSC2, while SSC1 only exists in chicken_1d and chicken_3w. *ACTC1*, *ID3*, *ID2*, *CYP2R1*, and *PROK2* were highly expressed in SSC1, while *KIT*, *DGKB*, *UCHL1*, *DMRT1* were highly expressed in SSC2 (Fig. 2C–D and Fig. S4A). Some key markers were also analyzed in each cluster of spermatocytes, and spermatids (Fig. S3C and D). Additionally, the total of 4,217, 13,498, and 4,347 potential markers and the molecular characteristics were explored for each cluster of spermatogonia spermatocytes, and spermatids (Fig. S4; Tables S5–7).

To identify key genes involved in spermatogenesis, we conducted a pseudotime analysis of germ cells via Monocle 2 (Fig. 2E; Table S8). The expression patterns of *DDX4*, *KIT*, *GOLGB1*, *FOXP2*, *HMGA2*, *DHRS4*, and *NRF1* suggested their important roles in the proliferation and differentiation of SSCs and Diff_ing S’gonia. Meiosis-related genes, including *RNF212*, *SPO11*, *HORMAD1*, and *SPATA22*, were highly expressed at the early stage of meiosis, and their expression decreased as meiosis progressed. *PGK2* has been reported to be essential for sperm function and male fertility [20]. *MYH9* and *CA2* were highly expressed in Pach and Dip, respectively. *SYCE3*, *DNAJC2*, *NME6*, *SYPL1*, *LDHA* and *MCM10* might play important roles in the development of RS. *CEP41* and *LDHB* were more highly expressed in the ES group, which could suggest their involvement in spermatogenesis (Fig. 2F; Fig. S3E). In pseudotime, we divided the spermatogenesis process into five stages based on the first 1,000 DEGs to explore the dynamic molecular characteristics of spermatogenesis. The expression of *ORC5*, *HMGA2*, *LAMB1*, *ASZ1*, *MAEL* and *DHRS4* increased, and genes related to stem cell population maintenance and differentiation increased in the first stage. *KMD5A*, *MYH9*, *YBX3*, *SYCE3*, *LCA5L* and *DNAJC2* were observed, and RNA splicing and chromosome organization were enriched in the second and third stages. *NME6*, *RPSH1*, *SPAG17*, *TTC26*, *RRP7A* and *TTLL1* were observed, and spermatid development was enriched in the fourth stage. *FRS2*, *IFT27*, *PLCZ1*, *CACNA2D1*, *CEP41* and *FOXM1* were observed, and cell differentiation occurred in the fifth stage (Fig. 2G). We also conducted pseudotime analysis of spermatogonia, spermatocytes, and spermatids to observe a large number of key pseudotime genes (Fig. S5; Tables S9–11).

To sum up, we conducted a systematic identification of chicken germ cells and studied the dynamic transcriptional characteristics of germ cell development, providing a wealth of data and molecular basis for in-depth research on the regulatory mechanism of spermatogenesis.

### The persistence of immature Sertoli cells in chicken testes after sexual maturity

**Fig. 3:**
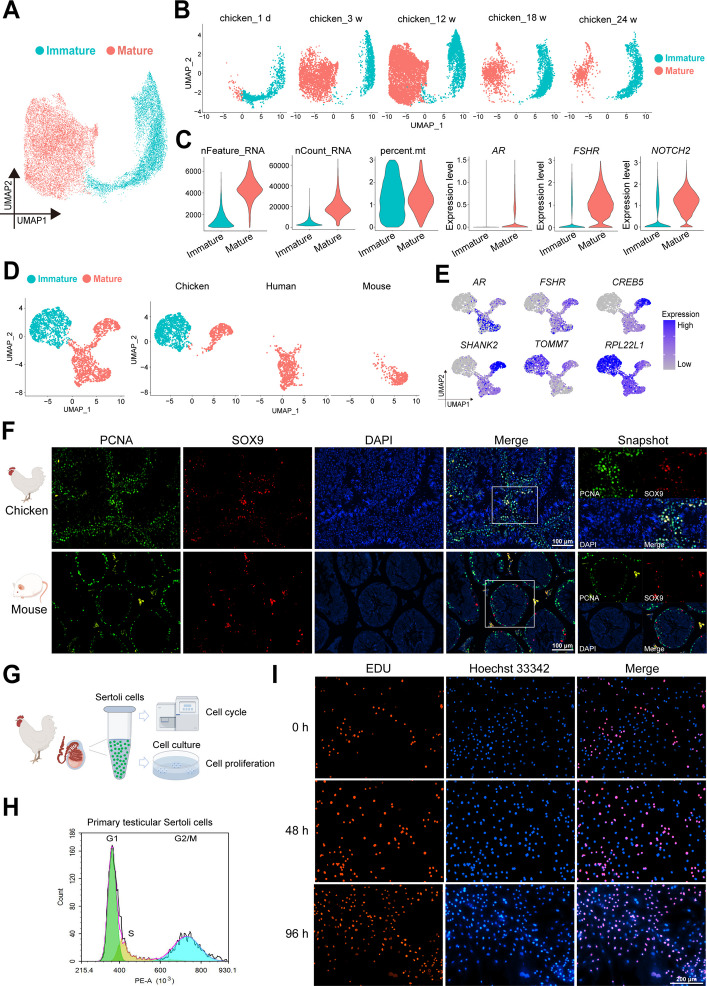
The persistence of immature Sertoli cells in chicken testes after sexual maturity. **A** A UMAP plot of testicular Sertoli cells of broiler chickens from five different groups (1 d, 3 weeks, 12 weeks, 18 weeks, and 24 weeks). Each dot represents a single Sertoli cell and is colored based on its cell subtype. **B** Identification of mature and immature Sertoli cells by analyzing nFeature_RNA, nCount_RNA, percent.mt and the expression patterns of classic marker genes. **C** Five UMAP plots showing the annotated types of Sertoli cells from five groups (1 d, 3 weeks, 12 weeks, 18 weeks, and 24 weeks), respectively. Each dot represents a single testicular cell and is colored based on its cell type. **D** UMAP plot of testicular Sertoli cells from chicken_24w, adult humans and adult mice. Each dot represents a single Sertoli cell and is colored based on its cell subtype. **E** Marker expression patterns of mature or immature Sertoli cells on UMAP plots. A gradient of blue and gray represents high or low marker expression levels. **F** Immunofluorescence staining for PCNA (green), SOX9 (red, a marker of Sertoli cells) and DAPI (blue) in testes of adult mice and chicken_24w. Scale bars = 100 μm. **G** Schematic of Sertoli cell extraction and experimental process. **H** Detection of primary Sertoli cell cycle in broiler chicken testis by flow cytometry. **I** Proliferation of testicular Sertoli cells of broiler chickens was measured using the EDU incorporation assay. EDU detection was performed at 0 h, 48 h, and 96 h of Sertoli cell adhesion, respectively. Red fluorescence represents EDU-labeled Sertoli cells. Nuclei were stained with Hoechst 33,342 (blue). Scale bars = 200 μm

Sertoli cells are indispensable in the process of spermatogenesis, and the number of Sertoli cells directly affects the production of sperm [12, 21]. Thus, we integrated the Sertoli cell cluster and found two main cell clusters as mature and immature Sertoli cells in chicken testes (Fig. 3A–C). Additionally, we identified 1,208 markers in immature Sertoli cells and 2,295 markers in mature Sertoli cells (Fig. S6A; Table S12). GO analysis revealed that immature Sertoli cells were enriched in “translation”, “oxidative phosphorylation”, “ribosome biogenesis”, “mitochondrion organization” and “metabolic process”, while mature Sertoli cells were enriched in “development process”, “protein modification process”, “intracellular signal transduction”, “regulation of response to stimulus” and “RNA biosynthetic process” (Fig. S6B).

Interestingly, we found that immature Sertoli cells persisted in the testes, even after sexual maturity, when analyzing Sertoli cells in chicken testes at five different periods (Fig. 3B). However, it is well known that immature Sertoli cells develop into mature Sertoli cells to provide energy for germ cells, and testicular Sertoli cells exist in a mature form and no longer proliferate after sexual maturity or adolescence in mammals [14]. To investigate the differences in the Sertoli cells of chicken and mammals, we integrated testicular Sertoli cells from chicken_24 weeks, adult human and adult mouse and identified two cell subtypes, namely, immature (*TOMM7*^+^, *RPL22L1*^+^) and mature (*AR*^+^, *FSHR*^+^, *CREB5*^+^, *SHANK2*^+^) Sertoli cells (Fig. 3D and E). We also found that both immature and mature Sertoli cells were present in chicken testes after sexual maturity, while only mature Sertoli cells were present in human and mouse testes.

Next, we detected the proliferation of testicular Sertoli cells in 28-week-old chickens and adult mice to verify the presence of immature Sertoli cells. Immunofluorescence co-staining of the proliferation marker PCNA and the Sertoli cell marker SOX9 revealed that there were proliferating Sertoli cells in the testes of the chickens at 24 weeks, but no co-stained cells were detected in the testes of the adult mice (Fig. 3F). Subsequently, we extracted and purified testicular Sertoli cells from chicken_24w to verify the presence of immature Sertoli cells in vitro (Fig. 3G). We detected the cell cycle distribution of the purified Sertoli cells by flow cytometry and found that 15.15% of the Sertoli cells were in the S phase and 34.93% were in the G2/M phase (Fig. 3H). Additionally, we cultured the extracted and purified Sertoli cells and tested their proliferation status by EdU staining. We observed EDU-positive Sertoli cells, and the number of these cells significantly increased with cultivation time (Fig. 3I). These active proliferation characteristics of immature Sertoli cells were consistent with previous studies [22]. Taken together, these results suggested that proliferating immature Sertoli cells persist in chicken testes after sexual maturity, which demonstrated the uniqueness of Sertoli cell development in chicken testes.

### CREB5 played a crucial role in the maturation of chicken Sertoli cells

**Fig. 4:**
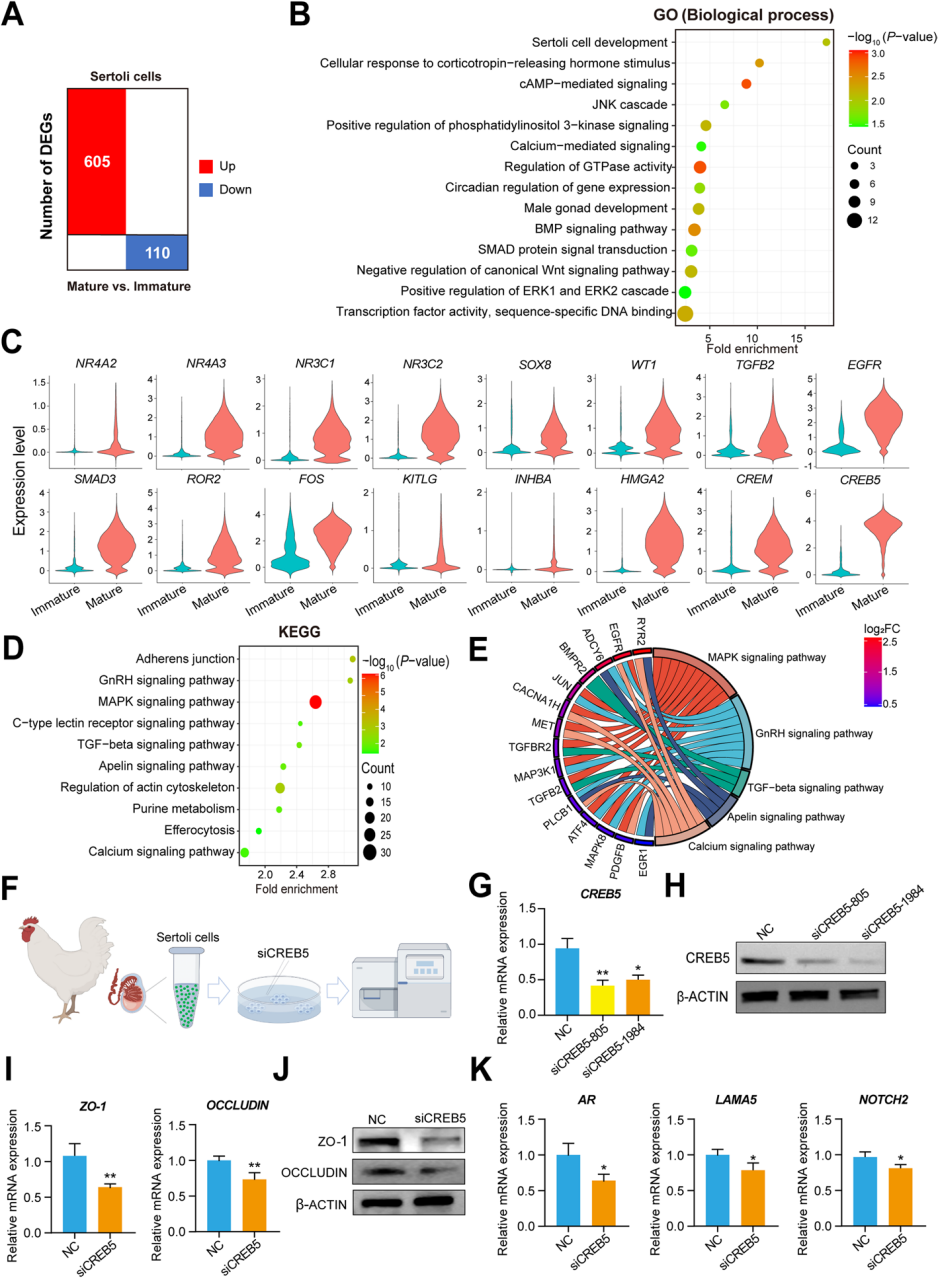
CREB5 played a crucial role in the maturation of chicken Sertoli cells. **A** Statistical analysis of the number of differentially expressed genes between mature and immature Sertoli cells. Red represents the number of upregulated genes, and blue represents the number of downregulated genes. **B** A dot bubble chart showing representative GO terms enriched in differentially expressed genes. **C** Expression patterns of representative genes of mature and immature Sertoli cells. **D** A dot bubble chart showing representative KEGG pathways enriched in differentially expressed genes. **E** A chord plot showing the subordinate relationship between representative genes (Left) and KEGG pathways (Right). The color of the boxes in front of the gene names from blue to red represents the fold change in gene expression. **F** Schematic of Sertoli cell extraction and *CREB5* interference. **G** and **H** The mRNA and protein expression of CREB5. **I** and **J** The mRNA and protein expression of ZO-1, and occludin in chicken Sertoli cells (*n* = 3). **K** The mRNA expression of *AR*, *LAMA5*, *NOTCH2* in chicken Sertoli cells (*n* = 3). ^*^P < 0.05, ^**^P < 0.01

To explore the maturation mechanism of Sertoli cells, we analyzed single-cell data from immature and mature Sertoli cells. Compared with those in immature Sertoli cells, we discovered 715 DEGs in mature Sertoli cells, including 605 upregulated genes and 110 downregulated genes (Fig. 4A; Table S13). Mature Sertoli cells were enriched in “Sertoli cell development”, “male gonad development”, “BMP signaling pathway”, “SMAD protein signal transduction”, “cellular response to corticotropin-releasing hormone stimulus” and some GO terms related to development and proliferation (Fig. 4B). Subsequently, we characterized some representative genes in these GO terms, such as *SOX8*, *TGFB2*, *EGFR*, *ROR2*, *NR4A2*, *NR4A3*, *NR3C1* and *NR3C2*. Notably, mature Sertoli cells also exhibited “transcription factor activity, sequence-specific DNA binding”, and the expression levels of *CREB5*, *CREM*, *WT1*, and *SMAD3* were significantly increased in mature Sertoli cells (Fig. 4C). KEGG analysis revealed that Sertoli cell maturation was associated with the “GNRH signaling pathway”, “MAPK signaling pathway”, “TGF-β signaling pathway”, “Apelin signaling pathway” and “Calcium signaling pathway”, and several related genes were characterized, including *RYR2*, *ADCY6*, *BMPR2*, *JUN*, *CACNA1H*, *MET*, *MAPK8*, *MAP3K1*, and *EGR1* (Fig. 4D and E). MAPK signaling pathway, TGF-β signaling pathway, and GnRH signaling pathway were crucial for Sertoli cell maturation [23–25].

Combining *CREB5* as the gene with the greatest differential expression, we hypothesized that *CREB5* might be involved in Sertoli cell maturation. We extracted and purified Sertoli cells from chicken testes, and conducted cell transfection using siCREB5 to detect the effect of *CREB5* on Sertoli cell function (Fig. 4F). The results showed that siCREB5 treatment decreased the mRNA and protein expression of CREB5 and siCREB5-1984 was used for future research (Fig. 4G and H). We found that siCREB5 treatment also decreased the mRNA and protein expression of blood-testis barrier (BTB) related genes ZO-1 and occludin (Fig. 4I and J). Additionally, siCREB5 decreased the mRNA expression of mature related markers *AR*, *LAMA5*, and *NOTCH2* (Fig. 4K). Our results suggested that *CREB5* might play a crucial role in maintaining the maturation of chicken Sertoli cells, laying the foundation for studying the mechanisms Sertoli cell maturation.

### Circadian rhythms participated in the development of Leydig cells in chicken testes

**Fig. 5:**
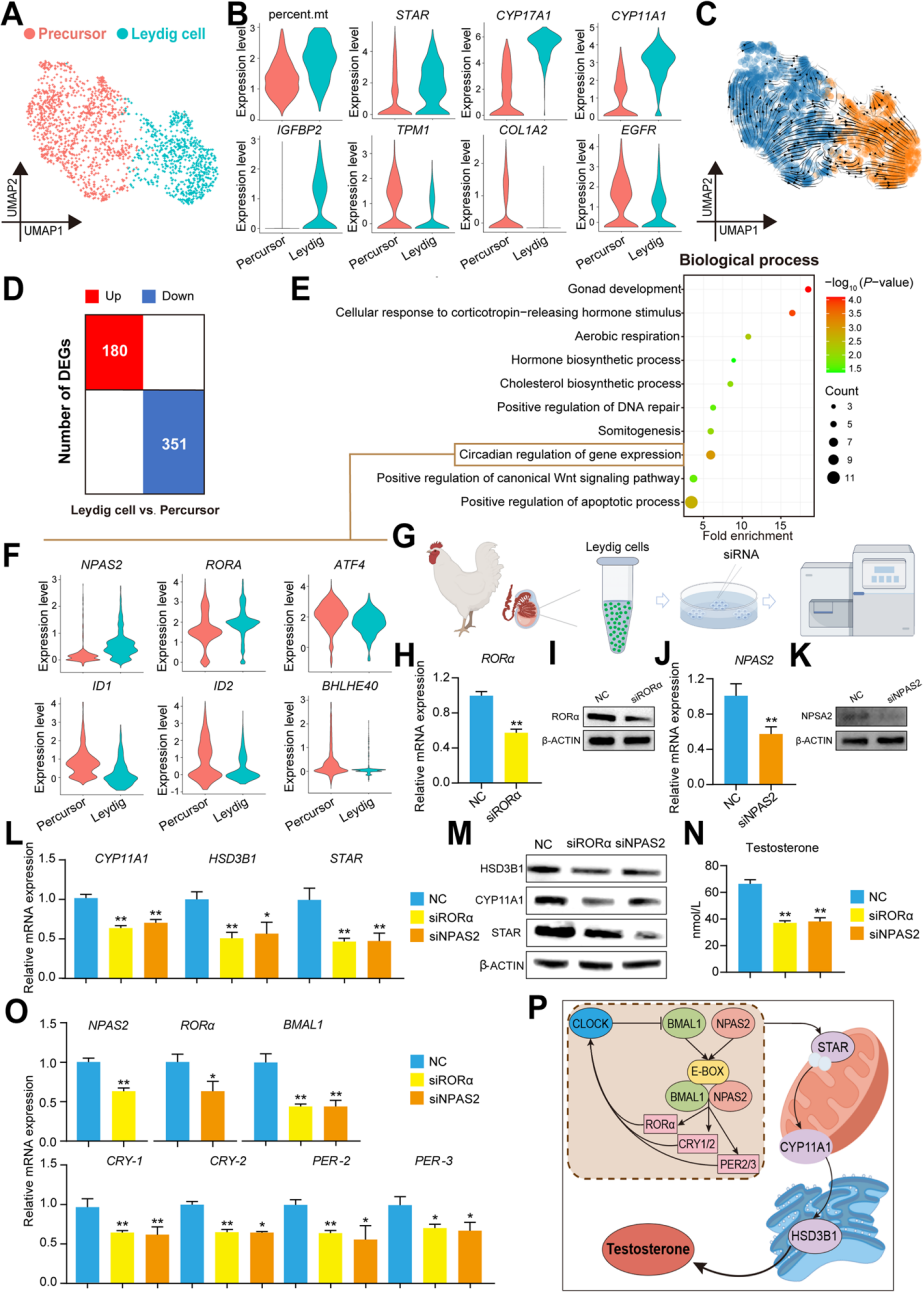
Circadian rhythm participated in the development of Leydig cells in chicken testes. **A** A UMAP plot of testicular Leydig cells of broiler chickens from five different groups (1 d, 3 weeks, 12 weeks, 18 weeks, and 24 weeks). Each dot represents a single Leydig cell and is colored based on its cell type. **B** Identification of precursor and Leydig cells by analyzing percent.mt and the expression patterns of classic marker genes. **C** RNA velocity analysis showing the dynamic change trend of precursor (blue) and Leydig (yellow) cells. **D** Statistical analysis of the number of differentially expressed genes between precursor and Leydig cells. Red represents the number of upregulated genes, and blue represents the number of downregulated genes. **E** A dot bubble chart showing representative GO terms enriched in differentially expressed genes. **F** Expression patterns of enriched genes in “circadian regulation of gene expression”. **G** Schematic of Leydig cell extraction and experimental process. **H**–**K** Relative mRNA and protein expression of *RORα* and *NPAS2* in Leydig cells (*n* = 3). **L** and **M** The mRNA and protein expression of key genes related to testosterone secretion, including CYP11A1, HSD3B1, and STAR in Leydig cells (*n* = 3). **N** Measurement of testosterone level in Leydig cells using ELISA (*n* = 3). **O** Relative mRNA expression of key genes related to circadian rhythm, including *NPAS2BMAL1*, *RORα*, *CRY-1*, *CRY-2*, *PER-2*, *PER-3* (*n* = 3). **P** Schematic diagram of circadian rhythm promoting testosterone secretion in chicken Leydig cells. ^*^P < 0.05, ^**^P < 0.01

Leydig cells can synthesize and secrete testosterone, which is particularly important for testicular development and spermatogenesis [6]. Thus, we integrated and analyzed the Leydig cell cluster and identified two main cell clusters (precursor and Leydig cells) by the expression patterns of classic marker genes (Fig. 5A and B). Specifically, the proportion of mitochondrial genes significantly increased, as mitochondria are the main site for testosterone secretion, and of course, genes related to testosterone secretion, including *STAR*, *CYP17A1*, and *CYP11A1*, were upregulated (Fig. 5B). Next, we calculated the markers of each cell cluster and identified 781 markers in precursor cells (*C7*, *EDNRA*, *APOA1*, *BTG2*, and *COL3A1*) and 420 markers in Leydig cells (*CYP17A1*, *CYP11A1*, *TPM2*, *LHCGR*, and *ND3*) (Fig. S6C; Table S14). GO analysis of the markers revealed that precursor cells were enriched in “cellular developmental process”, “cell population proliferation”, “RNA biosynthetic process”, “mesenchyme development” and “apoptosis process”, while Leydig cells were enriched in “cellular respiration”, “steroid biosynthetic process”, “generation of precursor metabolites and energy”, “lipid biosynthetic process” and “cholesterol metabolic process”, which are known functions of Leydig cells (Fig. S6D).

Importantly, the trend of cell differentiation from cell velocity analysis confirmed the developmental relationship of precursor and Leydig cells (Fig. 5C). Additionally, we found that some marker genes of precursor cells, such as *TPM1*, *APOA1*, *COL1A2*, and *COL3A1*, were highly expressed in TPCs. The results of UMAP and RNA velocity analysis also indicated that precursor cells could develop from TPCs. These results suggested that TPCs and Leydig cells might have the same source, as it has been reported in human testes that there is a common progenitor for peritubular myoid cells and Leydig cells [26].

To investigate the development mechanisms of Leydig cells, we compared precursor cells and identified 531 DEGs in Leydig cells, including 180 upregulated genes and 351 downregulated genes (Fig. 5D; Table S15). GO analysis of the markers indicated that Leydig cells were enriched in “gonad development”, “cellular response to corticotropin-releasing hormone stimulus”, “aerobic respiration”, “positive regulation of DNA repair”, and “cholesterol biosynthetic process” (Fig. 5E). The molecular characteristics of Leydig cell maturation are similar to those of human and mouse studies, both of which are related to steroid biological processes as well as energy metabolism [10, 27].

Interestingly, “circadian regulation of gene expression” was significantly enriched, and the expression levels of the neuronal PAS domain protein 2 (*NPAS2*) and the retinoic acid receptor-related orphan receptor-alpha (*RORα*) increased in Leydig cells, which might be closely related to testosterone secretion and the development of Leydig cells (Fig. 5F). Therefore, we demonstrated this in vitro by extracting testicular Leydig cells and treating them with siRORα and siNPAS2 (Fig. 5G). siRORα and siNPAS2 treatment significantly inhibited the relative mRNA and protein expression of RORα and NPAS2 (Fig. 5H–K). Notably, siRORα and siNPAS2 treatment significantly downregulated the relative mRNA and protein expression of CYP11A1, HSD3B1, and STAR, and decreased testosterone secretion in chicken Leydig cells (*P* < 0.01) (Fig. 5L–N). Additionally, we found that the relative mRNA expression of *RORα*, *NPAS2*, *BMAL1*, *CRY-1*, *CRY-2*, *PER-2*, and *PER-3* decreased significantly after *RORα* and *NPAS2* interference (*P* < 0.05) (Fig. 5O). The above results suggested that circadian rhythm could participated in testosterone secretion and the development of Leydig cells in chicken testes, which provided a new perspective for studying the development of Leydig cells (Fig. 5P).

### The dominant proportion of chicken Sertoli cells revealed by cross-species analysis

The sperm density of chickens is much greater than that of adult humans and mice, which suggests a unique system during spermatogenesis in chickens. Multi species analysis has been applied to explore the conservation and differences among species [28]. To characterize the spermatogenesis of chickens and mammals, we downloaded single-cell transcriptome data of adult human testes (*n* = 5; 16,015 cells) and adult mouse testes (*n* = 2; 5,264 cells) for cross-species analysis of chicken_24 weeks testes (*n* = 3; 23,893 cells). We used UMAP to integrate and analyze testicular cells from chickens, humans and mice.

**Fig. 6:**
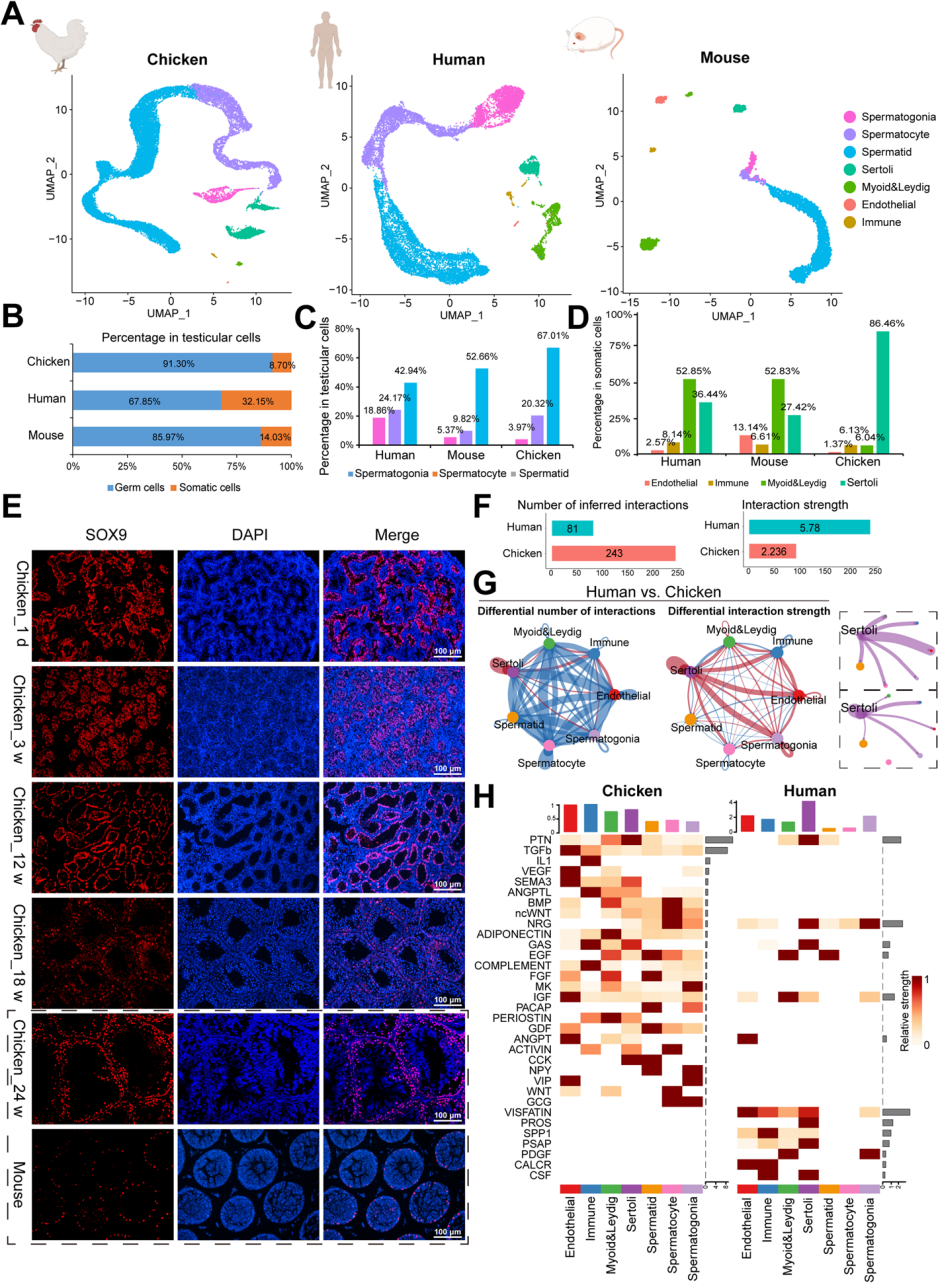
The dominant proportion of chicken Sertoli cells revealed by cross-species analysis. **A** UMAP plots of the annotated testicular cell types from chicken_24w (*n* = 3), adult human (*n* = 5), and adult mouse (*n* = 2). Each dot represents a single testicular cell and is colored based on its cell type. The single-cell transcriptome data of adults and mice are both from ArrayExpress with the accession codes E-MTAB-11063 (human) and E-MTAB-11071 (mouse). **B** A bar chart showing ratio of testicular germ cells and somatic cells in chicken_24w, adult human, and adult mouse. **C** A column chart showing ratio of spermatogonia, spermatocytes and spermatids in chicken_24w, adult human, and adult mouse. **D** A column chart showing ratio of endothelial cells, immune cells, myoid & Leydig cells, and Sertoli cells testicular somatic cells of chicken_24w, adult human, and adult mouse. **E** Immunofluorescence staining for SOX9 (red, a marker of Sertoli cells) and DAPI (blue) in testes of five-stage chicken (1 d, 3 weeks, 12 weeks, 18 weeks, and 24 weeks), and adult mouse. Scale bars = 100 μm. **F** Bar charts showing the number of inferred interactions and interaction strength of chicken_24w and adult human. **G** Circle plots (by CellChat analysis) showing the differential cell–cell communication network between chicken_24w and adult human, including the number of inferred interactions (left) and interaction strength (right). Circle plots in dashed boxes showing the number of inferred interactions between Sertoli cells and other cells. **H** Heatmaps showing the overall signaling patterns of each cell type in chicken_24w and adult human. The color of the small grid from light to dark indicates its relative strength

According to known specific marker genes of each cell type, we identified 7 major types of germ cells and somatic cells, including spermatogonia, spermatocytes, spermatids, Sertoli cells, endothelial cells, immune cells, and myoid & Leydig cells (Fig. 6A). To characterize the cellular composition of the three types of testicles, we calculated the proportions of each cell type. Our results revealed that the proportion of testicular germ cells in chicken, which accounted for 91.30%, was much greater than that in human (85.97%) and mice (67.85%) (Fig. 6B). We observed particularly efficient spermatogenesis, with spermatids accounting for up to 67.01% of the total number of testicular germ cells in chickens (42.94% in humans and 52.66% in mice) (Fig. 6C).

Additionally, we found that the composition proportion of somatic cells in the microenvironment of chicken testes was significantly different from that of humans and mice. The proportion of chicken Sertoli cells in testicular somatic cells was as high as 86.48%, which is much higher than that in humans (36.44%) and mice (27.42%), while myoid and Leydig cells made up the greatest proportion of testicular somatic cells in humans and mice (Fig. 6D). Our results reflected the specificity and differences in the composition of chicken testicular cells. Simultaneously, the fluorescence staining results of SOX9 revealed the distribution of Sertoli cells in chicken testes, and compared with that in mouse testes, more chicken testicular Sertoli cells were distributed in the seminiferous tubules (Fig. 6E).

We then investigated the interactions between somatic cells and germ cells in chicken and human testes via CellChat analysis. Interestingly, the number of inferred interactions in chickens was three times that in humans, although the intensity was lower than that in humans (Fig. 6F). In particular, there were significant differences in the number of interactions and interaction strength between chicken and human testicular Sertoli cells (Fig. 6G). Next, we analyzed the overall signaling patterns in the cell‒cell communication network between chicken and human testes. Overall, there were more signaling patterns in chicken testes than in human testes, such as TGF-β, SEMA3, ANGPTL, BMP, ncWNT, GAS, EGF, MK, periostin, GDF, ANGPT, activin, and CCK signaling in Sertoli cells (Fig. 6H). Taken together, these results demonstrated the uniqueness of Sertoli cell development in chicken testes.

## Discussion

It is important to investigate the molecular features of spermatogenesis for understanding the differences and conservatism of testicular development across different species [1, 16, 28]. In this study, through establishing a single-cell transcriptome profile of chicken testes from hatching to maturity, we identified the dynamic transcriptional characteristics of germ cell fate transition and explored the unique adaptations of Sertoli cells in chicken testes. Most importantly, we demonstrated that immature Sertoli cells persist in chicken testes after sexual maturity, contrasting with the known development of Sertoli cells in mammals. We also elucidated the developmental characteristics of Sertoli cells and Leydig cells. These results may supplement the theory of testicular Sertoli cell development and hold extraordinary significance in understanding the unique adaptations in poultry.

### Cell recognition

Cell recognition is a fundamental and important step in single-cell data analysis [29]. We identified 11 main cell types, including 8 types of somatic cells and 3 types of germ cells. In addition to nerve cells, other types of testicular cells and their markers have been proven by many single-cell testicular articles [26, 30–32]. Notably, we observed nerve cells in chicken testes, which was confirmed by marker identification and GO analysis. Previous studies have shown that the nerves distributed in the testes, originating from the spermatic plexus, are composed of many small nerve fibers from the renal plexus and abdominal aortic plexus, and these nerve fibers are unmyelinated and descend along the testicular artery, known as the superior spermatic nerves [33, 34]. Testicular nerves reportedly play an important role in the regulation of testosterone secretion [35].

Additionally, we identified SSC1 and SSC2 in Undiff_ ed S'gonia, which have different gene expression patterns. *ACTC1* served as a marker of early first trimester human trophoblast in human embryonic stem cell model [36]. *ID3*, *ID2*, *CYP2R1*, and *PROK2* have also reported to participate in testicular development [37–39]. *UCHL1* promoted SSCs self-renewal, while *KIT* and *DMRT1* are considered as spermatogonial proliferation signals [40, 41]. These results suggested that SSC1 might be closer to primitive stem cells, and SSC2 could self-renew and differentiate into Undiff_ing S’gonia according to the developmental trajectory. Differently, the single-cell transcriptome study of human testes identified five discrete transcriptional/developmental spermatogonial states, including a new early SSC state (state 0), and identified significant similarities between adult state 0 and infant SSC [19].

### Proliferating Sertoli cells persist in chicken testes after sexual maturity

Sertoli cells play a central role in testicular development and spermatogenesis, which has also been proven in single-cell transcriptome studies of human azoospermia [13, 21, 42]. Numerous pieces of evidence indicate that Sertoli cell number directly determines the testamentary size and sperm production [12]. In our study, we revealed the unique adaptations in chicken Sertoli cells. The most important was the persistent presence of proliferating Sertoli cells in chicken testes after sexual maturity, which were confirmed by the immunofluorescence results of co-staining with PCNA and SOX9, as well as cell cycle distribution and EDU positive labeling of chicken Sertoli cells. Conversely, immature Sertoli cells mature during puberty and are no longer proliferating after puberty in mammalian testes [43]. The sustained existence of immature Sertoli cells can develop into mature Sertoli cells to continuously provide nutrition and exert phagocytic function during spermatogenesis [44]. A previous report provided strong evidence that transplanting mouse fetal testicular cells into a Sertoli-depleted adult testis can promote the colonization of mouse fetal Sertoli cells in adult seminiferous tubules and support host testicular spermatogenesis, which may suggest that immature fetal Sertoli cells can become sufficient maturation and functional in the adult testis. [45]. These results reflected the sustained presence of proliferating Sertoli cells in chicken testes after sexual maturity, which might be one of the reasons for the high sperm density.

### CREB5 participates in maintaining the maturation and function of chicken Sertoli cells

The maturation of Sertoli cells is accompanied by significant changes in gene expression [44]. We found that the expression levels of *AR*, *FSHR*, and *CREB5* increased in mature Sertoli cells. It has been reported that *AR* is a marker of Sertoli cell maturation, and its expression changes during the spermatogenic cycle [44, 46]. *FSHR*, a marker of Sertoli cell maturation regulation, binds to FSH and stimulates cAMP to control the proliferation, development, and function of Sertoli cells [47, 48]. *CREB5*, as a binding protein of cAMP, has also been reported to be associated with resistance to AR-targeted therapy [49], which may become a new marker of chicken testicular Sertoli cells. Importantly, our results indicated that *CREB5* interference reversed the maturation characteristics of Sertoli cells and disrupted the structure of BTB. A previous study has shown that *CREB5* promotes joint formation and the subsequent development of articular chondrocytes, which suggesting the importance of *CREB5* for organ and cell development [50]. Taken together, our results indicated that *CREB5* participates in maintaining the maturation and function of chicken Sertoli cells.

### Circadian rhythm promotes testosterone secretion in Leydig cells

Leydig cells are the main site for testosterone synthesis and secretion, and testosterone is indispensable during spermatogenesis [51]. Our results indicated that the development of Leydig cells was closely related to the circadian rhythm in chicken testes, accompanied by the upregulation of *NPAS2* and *RORα* expression. It is widely known that the photoperiod is particularly important for regulating chicken production and reproduction [52]. There is limited direct evidence that circadian rhythms are involved in regulating testosterone secretion and the development of Leydig cells [53–55]. *RORα*, as an important circadian clock gene has been reported to regulate testosterone secretion of Leydig cells [56]. *NPAS2* is an important regulatory factor that generates and maintains periodic rhythms, regulating various physiological functions such as sleep, arousal, endocrine, metabolism, cell proliferation and apoptosis, and immunity [57]. It has been reported that *NPAS2* forms a heterodimer with *BMAL1*, which binds to E-box enhancer elements, thereby promoting the transcriptional activation of circadian rhythm-related genes, such as *RORα*, *CRY1/2* and *PER2/3* [58]. However, there is no direct evidence to indicate the role of *NPAS2* in regulating testosterone secretion and the development of Leydig cells. In our study, *RORα* and *NPAS2* interference decreased the expression of circadian rhythm related genes *BMAL1*, *CRY-1*, *CRY-2*, *PER-2*, and *PER-3* and inhibited testosterone secretion, accompanied by a decrease in the expression of *STAR*, *CYP17A1*, and *CYP11A1*. Taken together, these results suggested that circadian rhythm participated in testosterone secretion and the development of Leydig cells in chicken testes.

### Multispecies analysis of key signaling between chicken testicular cells

Quoting single-cell testicular data from other species from databases has been widely applied for cross species analysis [59]. Compared to adult humans and mice, cross-species analysis revealed a greater proportion of germ cells in mature chickens, especially spermatids. In a previous study on single-cell testes involving species evolution, the proportion of testicular cells in various species was statistically analyzed, indicating that the proportion of germ cells in red jungle fowl was higher than that in mammals, including humans, mice, monkeys, and platypus [16]. These results of high proportion of germ cells were an important foundation for the formation of high sperm density in chickens.

The cellular interactions in chicken testes were three times greater than those in human testes, mainly involving TGF-β signaling, VEGF signaling, BMP signaling, FGF signaling and activin signaling. We also found the specific activation of Sertoli cell interaction signals, such as TGF-β, BMP, EGF, and activin. Studies have reported that TGF-β signaling, VEGF signaling, BMP signaling, FGF signaling and their interactions participate in cell growth and organ development [60, 61]. FGF signaling acted upstream of RA signaling to activate the PI3K/Akt pathway in germ cells and played an important role in spermatogenesis [62, 63]. Activin signaling was involved in Sertoli cell differentiation and function, increased activin signaling induces the proliferation of primary Sertoli cells, and activin A reprograms Sertoli cells to immature and dedifferentiated states [64]. The above results reflected the key signaling of chicken spermatogenesis, which was particularly important for the formation of unique adaptations in chickens.

## Conclusion

In summary, we established a complete single-cell transcriptome profile of chicken testes from hatching to maturity, revealing the unique adaptations in chicken Sertoli cells. Most importantly, proliferating Sertoli cells persisted in chicken testes even after sexual maturity. We also observed the dominance of Sertoli cells in somatic composition of mature chicken testes, and the specific activation of Sertoli cell interaction signals. Moreover, our results indicated that *CREB5* played a crucial role in maintaining the maturation and function of chicken Sertoli cells, and circadian rhythm promoted testosterone secretion and the development of Leydig cells in chicken testes. These findings might lay a molecular foundation for elucidating the poultry spermatogenic superiority and provided new insights into the formation of high sperm density in poultry. The sample sizes of 1 d and 3 weeks (*n* = 2) may affect the reliability and statistical ability of the results, as well as the lack of more in-depth mechanism research, which may be a limitation of this study. In the future, we will delve into the key targets and molecular mechanisms underlying the formation of high sperm density.

## Supplementary Information

Additional file 1: Fig. S1. Quality control and information of scRNA-seq. A Quality of scRNA-seq of 13 broiler testicular samples, including nFeature_RNA, nCount_RNA and percent.mt. B Information summary of 13 broiler testicular samples sequenced in this study. C A UMAP plot showing the annotated testicular cell types. Each dot represents a single testicular cell and is colored based on the five different ages of broilers. D Thirteen UMAP plots showing the annotated testicular cell types of each broiler sample sequenced in this study with color based on the cell type. E Immunofluorescence staining for SOX9, ACTA2, VWF, CLEC3B, DDX4, TOP2A, TPPP2 (red) and DAPI (blue) in testes. Scale bars = 100 μm. Fig. S2. Marker expression and percentage of each cluster of five different ages. A Marker expression patterns of each cluster on UMAP plots. A gradient of blue and gray represents high or low marker expression levels. B Bar plot showing the percentage of each cluster of five groups (1 d, 3 weeks, 12 weeks, 18 weeks, and 24 weeks). Fig. S3. Dynamic transcriptional characteristics of germ cell development in chicken testes. A Bar plot showing the number of testicular germ cells and percentage of each cluster from five groups (1 d, 3 weeks, 12 weeks, 18 weeks, and 24 weeks). The dashed line represents spermatocytes, while the solid line represents spermatids. B Heatmap showing the markers of each cell cluster of germ cells by “DoHeatmap” function. All markers are calculated by “FindAllMarker” function. The number on the left shows the number of differentially expressed genes and is colored based on its cell type. C UMAP plots showing the annotated cell types of spermatocytes and spermatids. Dashed lines and arrows represent their developmental trajectory. D Heatmaps showing the representative markers of each cell cluster of spermatocytes and spermatids. A gradient of red and blue represents high or low marker expression levels. E Expression patterns of representative dynamic genes as predicted by pseudotime during spermatogenesis. Germ cells are colored based on the cluster colors and ordered according to the pseudotime trajectory. Pseudotime (SSCs to ES2) is indicated below each gene plot column. Fig. S4. Potential markers and molecular characteristics of spermatogonia, spermatocytes, and spermatids in chickens. A–C Heatmap showing the markers of each cell cluster of spermatogonia (A), spermatocytes (B), and spermatids (C). The number on the left shows the number of differentially expressed genes, and the right side shows top 5 markers. D–F Charts showing 5 representative GO terms enriched in the marker genes of each cluster of spermatogonia (D), spermatocytes (E), and spermatids (F) with −log_10_(*P*-value) of each GO term. Fig. S5. Pseudotime analysis of spermatogonia, spermatocytes, and spermatids. A–C Pseudotime trajectory of spermatogonia (A), spermatocytes (B), and spermatids (C) revealed cellular states based on the pseudotime. Each dot represents a single germ cell and is colored based on its cell subtype. D–F Heatmap showing the clusters of top 1,000 genes in pseudotime from spermatogonia (D), spermatocytes (E), and spermatids (F) of broiler chickens. A gradient of red and blue represents high or low gene expression levels based on the expression color code. The number on the left side shows the number of differentially expressed genes, and the right side shows the representative genes and GO terms. Fig. S6. Developmental analysis of Leydig cells and Sertoli cells. A Heatmap showing the markers of immature and mature Sertoli cells. The number on the left shows the number of differentially expressed genes, and the right side shows top 5 marker genes. B Bar chart showing 5 representative GO terms enriched in the marker genes of immature and mature Sertoli cells with −log_10_ (adjusted *P*-value) of each GO term. C Heatmap showing the markers of precursor and Leydig cells. The number on the left shows the number of differentially expressed genes, and the right side shows top 5 marker genes. D Bar chart showing 5 representative GO terms enriched in the marker genes of precursor and Leydig cells with −log_10_ (adjusted *P*-value) of each GO term

Additional file 2: Table S1. The sequences of siRNA and NC. Table S2. The primer sequences for RT-qPCR. Table S3. All markers of various cell types in chicken testes. Table S4. All markers of each type of germ cells in chicken testes. Table S5. All markers of each type of spermatogonia in chicken testes. Table S6. All markers of each type of spermatocytes in chicken testes. Table S7. All markers of each type of spermatids in chicken testes. Table S8. Top1000 genes in pseudotime of chicken germ cells. Table S9. Top1000 genes in pseudotime of chicken spermatogonia. Table S10. Top1000 genes in pseudotime of chicken spermatocytes. Table S11. Top1000 genes in pseudotime of chicken spermatids. Table S12. Markers of mature and immature Sertoli cells in chicken. Table S13. Markers of percursor and Leydig cells in chicken testes. Table S14. DEGs of mature Sertoli cells compared with immature in chicken testes. Table S15. DEGs of Leydig cells compared with percursor in chicken testes

## Data Availability

Raw and processed single-cell transcriptome data of 13 chicken testes at 1 d, 3 weeks, 12 weeks, 18 weeks, and 24 weeks have been deposited in NCBI with the BioProject codes PRJNA1119270. The analysis and experimental data in this study are also available from the corresponding author according to reasonable requirements.

## Abbreviations

*ALDH1A1*: Aldehyde dehydrogenase 1 family member A1
ANOVA: Analysis of variance
*AR*: Androgen receptor
*AVD*: Avidin
*BMAL1*: Brain and muscle arnt-like 1
*BMP*: Bone morphogenetic proteins
BTB: Blood-testis barrier
*CA2*: Carbonic anhydrase 2
*CDH5*: Cadherin 5
*CEP41*: Centrosomal protein 41
*CREB5*: cAMP responsive element binding protein 5
*CRY-1*: Cryptochrome 1
*CRY-2*: Cryptochrome 2
*CYP11A1*: Cytochrome P450 family 11 subfamily A member 1
*DDX4*: DEAD-box helicase 4
DEGs: Differential expressed genes
*DHRS4*: Dehydrogenase/reductase 4
*DNAJC2*: DnaJ heat shock protein family (Hsp40) member C2
*EGF*: Epidermal growth factor
ELISA: Enzyme-linked immunosorbent assay
*FOXM1*: Forkhead box M1
*FOXP2*: Forkhead box protein P2
GO: Gene Ontology
*GOLGB1*: Golgin B1
*GSTA2*: Glutathione S-transferase alpha 2
H&E: Hematoxylin and eosin
*HMGA2*: High mobility group AT-hook 2
*HORMAD1*: HORMA domain-containing 1
*HSD3B1*: Hydroxy-delta-5-steroid dehydrogenase, 3 beta- and steroid delta-Isomerase 1
*IFT27*: Phospholipase C zeta 1
KEGG: Kyoto Encyclopedia of Genes and Genomes
*KIT*: KIT proto-oncogene, receptor tyrosine kinase
*KMD5A*: Lysine demethylase 5A
*LAMB1*: Laminin subunit beta 1
*LCA5L*: Lebercilin-like protein
*LDHA*: Lactate dehydrogenase A
*LDHB*: Lactate dehydrogenase B
*MAEL*: Maelstrom spermatogenic transposon silencer
*MCM10*: Minichromosome maintenance 10 replication initiation factor
*MYH9*: Myosin heavy chain 9
*NME6*: NME/NM23 nucleoside diphosphate kinase 6
*NOTCH2*: Notch receptor 2
*NPAS2*: Neuronal PAS domain protein 2
*NRF1*: Nuclear respiratory factor 1
*ORC5*: Origin recognition complex subunit 5
PBS: Phosphate-buffered saline
PCA: Principal component analysis
*PCNA*: Proliferating cell nuclear antigen
*PER-2*: Period circadian regulator 2
*PER-3*: Period circadian regulator 3
PFA: Paraformaldehyde
*PGK2*: Phosphoglycerate kinase 2
*PLCZ1*: Intraflagellar transport 27
*RNF212*: Ring finger protein 212
*RORα*: Retinoic acid receptor-related orphan receptor alpha
RT-qPCR: Quantitative real-time PCR
S’gonia: Spermatogonia
scRNA-seq: Single-cell RNA sequencing
S'cyles: Spermatocytes
*SOX18*: SRY-box transcription factor 18
*SOX9*: SRY-box transcription factor 9
*SPAG17*: Sperm-associated antigen 17
*SPATA22*: Spermatogenesis-associated 22
*SPATA4*: Spermatogenesis associated 4
*SPO11*: SPO11 initiator of meiotic double-strand breaks
SSCs: Spermatogonial stem cells
*STAR*: Steroidogenic acute regulatory protein
*SYCE3*: Synaptonemal complex central element 3
*SYPL1*: Synaptophysin-like 1
*TGF-β*: Transforming growth factor-β
*TOP2A*: Topoisomerase II alpha
TPCs: Testicular peritubular cells
*TTC26*: Tetratricopeptide repeat domain 26
*TTLL1*: Tubulin tyrosine ligase-like 1
*TUBA8B*: Tubulin alpha 8b
*UMAP*: Uniform manifold approximation and projection
*VWF*: Von willebrand factor
*YBX3*: Y-box binding protein 3
*ZO-1*: Zonula occludens-1
