# Supplementary figures and images for: Unique Sertoli cell adaptations support enhanced spermatogenesis in chickens
Source: J Anim Sci Biotechnol. 2025 Dec 10;16:170. doi: 10.1186/s40104-025-01304-8 (PMC12690856; doi:10.1186/s40104-025-01304-8)

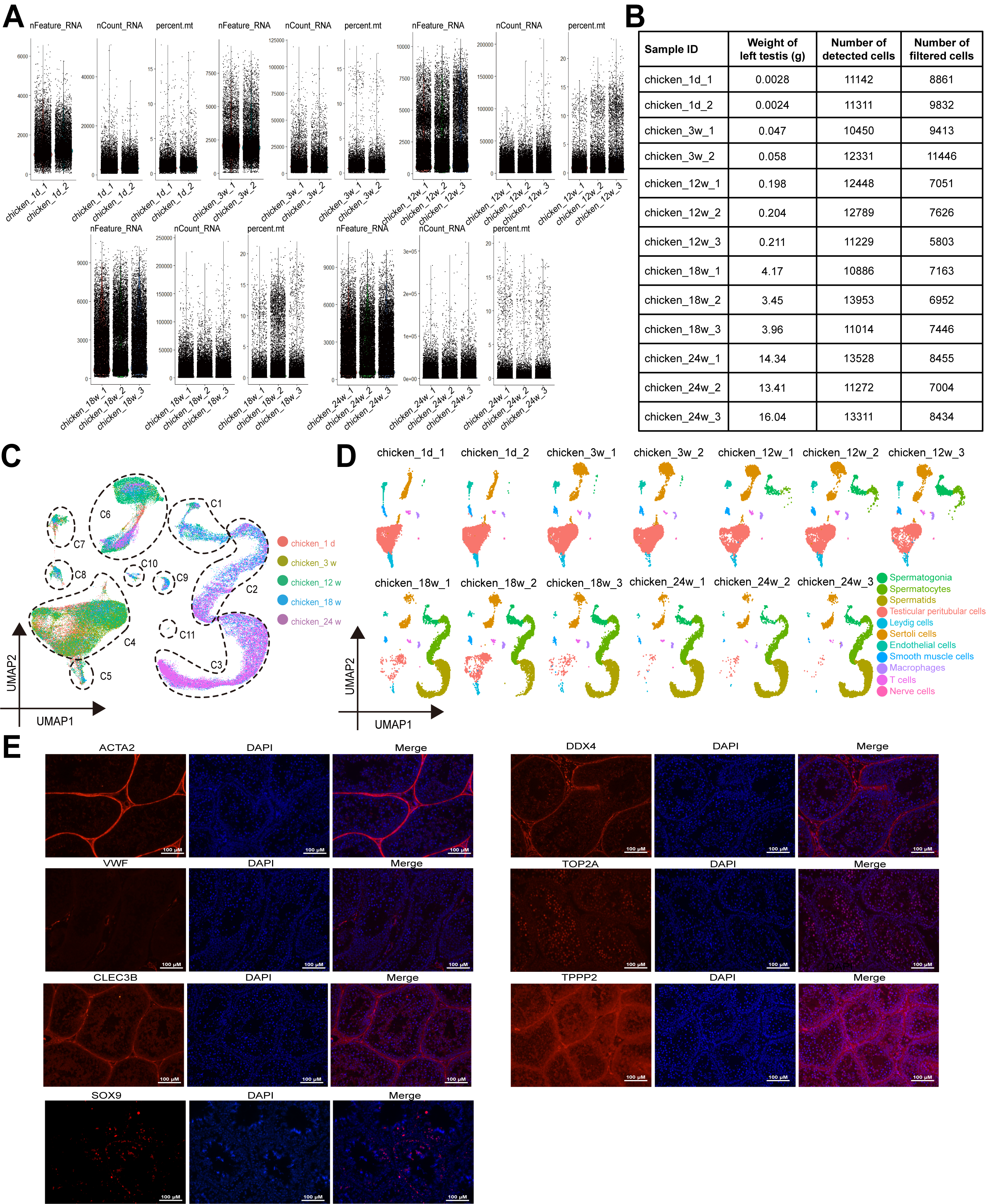

Supplement: Supplementary file 1 — Additional file 1: Fig. S1. Quality control and information of scRNA-seq. A Quality of scRNA-seq of 13 broiler testicular samples, including nFeature_RNA, nCount_RNA and percent.mt. B Information summary of 13 broiler testicular samples sequenced in this study. C A UMAP plot showing the annotated testicular cell types. Each dot represents a single testicular cell and is colored based on the five different ages of broilers. D Thirteen UMAP plots showing the annotated testicular cell types of each broiler sample sequenced in this study with color based on the cell type. E Immunofluorescence staining for SOX9, ACTA2, VWF, CLEC3B, DDX4, TOP2A, TPPP2 (red) and DAPI (blue) in testes. Scale bars = 100 μm. Fig. S2. Marker expression and percentage of each cluster of five different ages. A Marker expression patterns of each cluster on UMAP plots. A gradient of blue and gray represents high or low marker expression levels. B Bar plot showing the percentage of each cluster of five groups (1 d, 3 weeks, 12 weeks, 18 weeks, and 24 weeks). Fig. S3. Dynamic transcriptional characteristics of germ cell development in chicken testes. A Bar plot showing the number of testicular germ cells and percentage of each cluster from five groups (1 d, 3 weeks, 12 weeks, 18 weeks, and 24 weeks). The dashed line represents spermatocytes, while the solid line represents spermatids. B Heatmap showing the markers of each cell cluster of germ cells by “DoHeatmap” function. All markers are calculated by “FindAllMarker” function. The number on the left shows the number of differentially expressed genes and is colored based on its cell type. C UMAP plots showing the annotated cell types of spermatocytes and spermatids. Dashed lines and arrows represent their developmental trajectory. D Heatmaps showing the representative markers of each cell cluster of spermatocytes and spermatids. A gradient of red and blue represents high or low marker expression levels. E Expression patterns of representative dynami [file 40104_2025_1304_MOESM1_ESM.zip › Figure S1.tif]

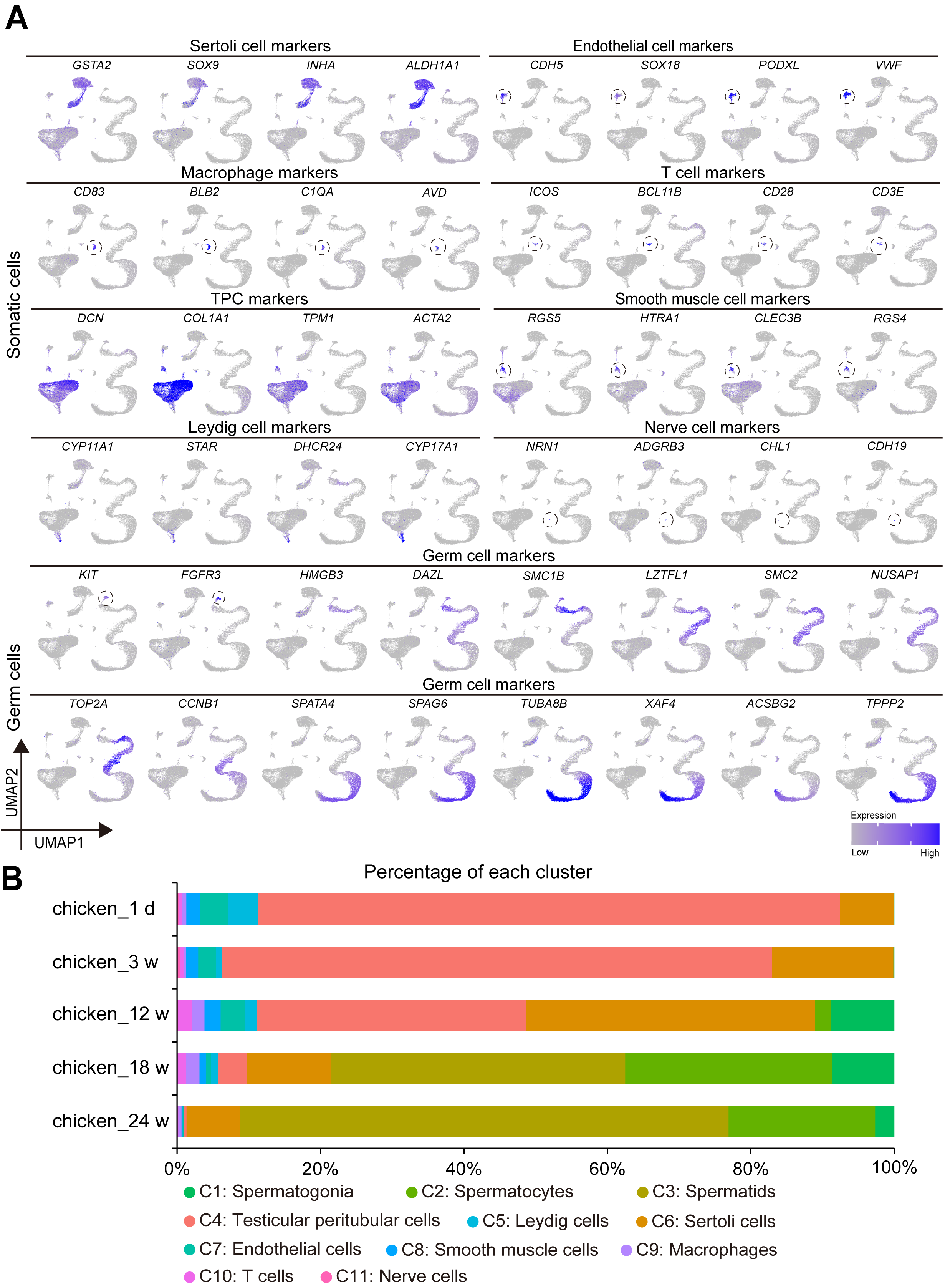

Supplement: Supplementary file 1 — Additional file 1: Fig. S1. Quality control and information of scRNA-seq. A Quality of scRNA-seq of 13 broiler testicular samples, including nFeature_RNA, nCount_RNA and percent.mt. B Information summary of 13 broiler testicular samples sequenced in this study. C A UMAP plot showing the annotated testicular cell types. Each dot represents a single testicular cell and is colored based on the five different ages of broilers. D Thirteen UMAP plots showing the annotated testicular cell types of each broiler sample sequenced in this study with color based on the cell type. E Immunofluorescence staining for SOX9, ACTA2, VWF, CLEC3B, DDX4, TOP2A, TPPP2 (red) and DAPI (blue) in testes. Scale bars = 100 μm. Fig. S2. Marker expression and percentage of each cluster of five different ages. A Marker expression patterns of each cluster on UMAP plots. A gradient of blue and gray represents high or low marker expression levels. B Bar plot showing the percentage of each cluster of five groups (1 d, 3 weeks, 12 weeks, 18 weeks, and 24 weeks). Fig. S3. Dynamic transcriptional characteristics of germ cell development in chicken testes. A Bar plot showing the number of testicular germ cells and percentage of each cluster from five groups (1 d, 3 weeks, 12 weeks, 18 weeks, and 24 weeks). The dashed line represents spermatocytes, while the solid line represents spermatids. B Heatmap showing the markers of each cell cluster of germ cells by “DoHeatmap” function. All markers are calculated by “FindAllMarker” function. The number on the left shows the number of differentially expressed genes and is colored based on its cell type. C UMAP plots showing the annotated cell types of spermatocytes and spermatids. Dashed lines and arrows represent their developmental trajectory. D Heatmaps showing the representative markers of each cell cluster of spermatocytes and spermatids. A gradient of red and blue represents high or low marker expression levels. E Expression patterns of representative dynami [file 40104_2025_1304_MOESM1_ESM.zip › Figure S2.tif]

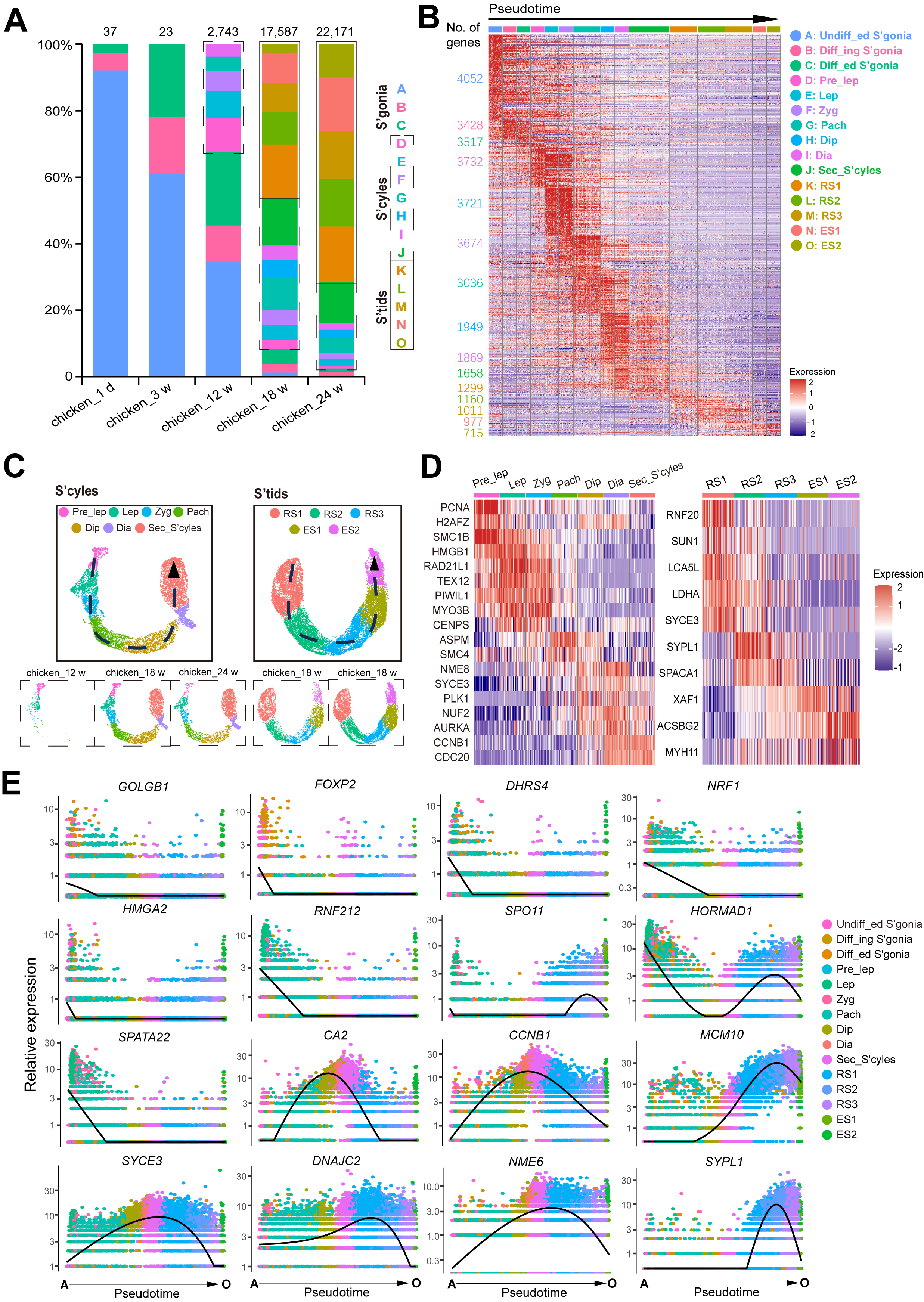

Supplement: Supplementary file 1 — Additional file 1: Fig. S1. Quality control and information of scRNA-seq. A Quality of scRNA-seq of 13 broiler testicular samples, including nFeature_RNA, nCount_RNA and percent.mt. B Information summary of 13 broiler testicular samples sequenced in this study. C A UMAP plot showing the annotated testicular cell types. Each dot represents a single testicular cell and is colored based on the five different ages of broilers. D Thirteen UMAP plots showing the annotated testicular cell types of each broiler sample sequenced in this study with color based on the cell type. E Immunofluorescence staining for SOX9, ACTA2, VWF, CLEC3B, DDX4, TOP2A, TPPP2 (red) and DAPI (blue) in testes. Scale bars = 100 μm. Fig. S2. Marker expression and percentage of each cluster of five different ages. A Marker expression patterns of each cluster on UMAP plots. A gradient of blue and gray represents high or low marker expression levels. B Bar plot showing the percentage of each cluster of five groups (1 d, 3 weeks, 12 weeks, 18 weeks, and 24 weeks). Fig. S3. Dynamic transcriptional characteristics of germ cell development in chicken testes. A Bar plot showing the number of testicular germ cells and percentage of each cluster from five groups (1 d, 3 weeks, 12 weeks, 18 weeks, and 24 weeks). The dashed line represents spermatocytes, while the solid line represents spermatids. B Heatmap showing the markers of each cell cluster of germ cells by “DoHeatmap” function. All markers are calculated by “FindAllMarker” function. The number on the left shows the number of differentially expressed genes and is colored based on its cell type. C UMAP plots showing the annotated cell types of spermatocytes and spermatids. Dashed lines and arrows represent their developmental trajectory. D Heatmaps showing the representative markers of each cell cluster of spermatocytes and spermatids. A gradient of red and blue represents high or low marker expression levels. E Expression patterns of representative dynami [file 40104_2025_1304_MOESM1_ESM.zip › Figure S3.tif]

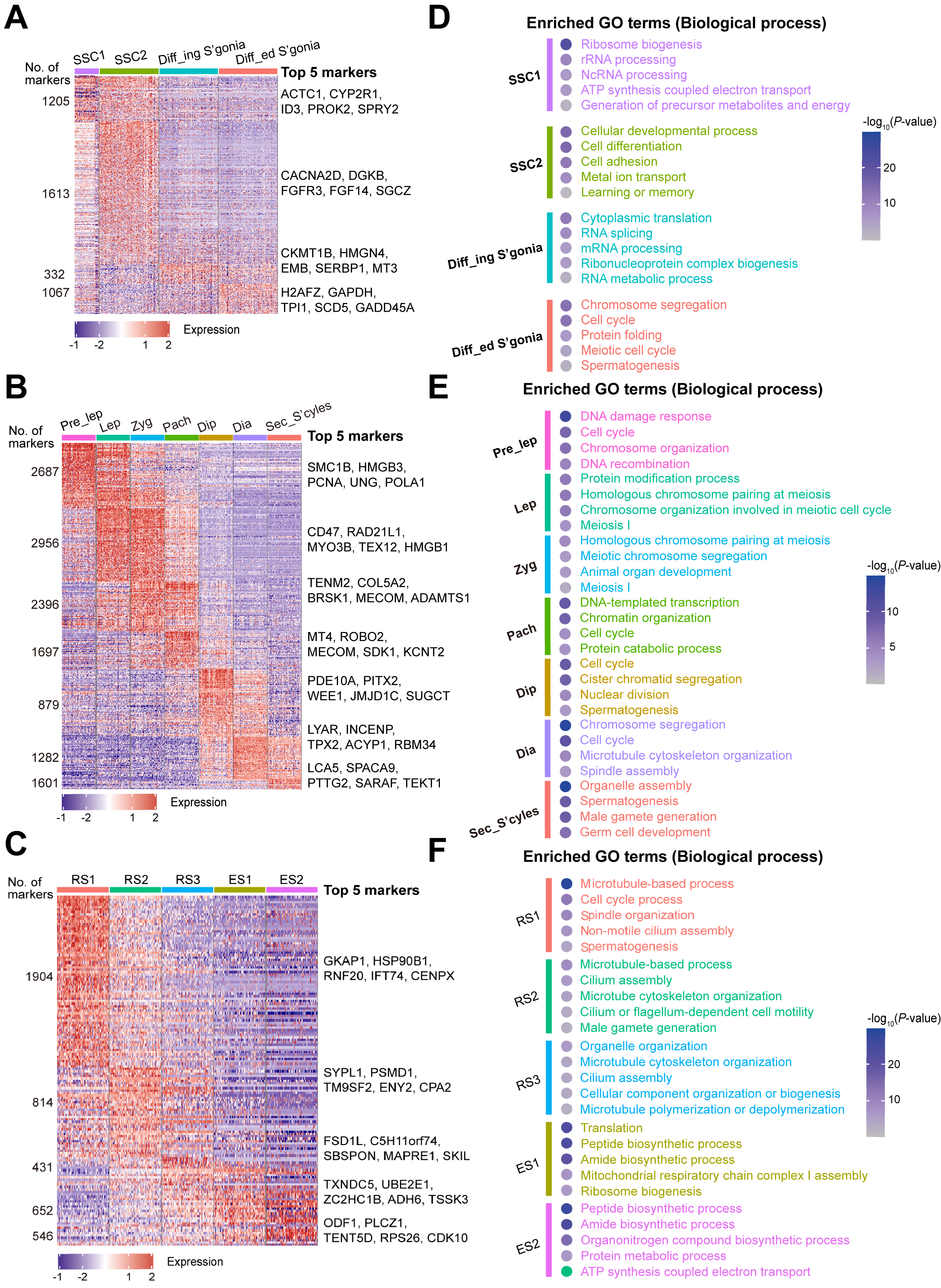

Supplement: Supplementary file 1 — Additional file 1: Fig. S1. Quality control and information of scRNA-seq. A Quality of scRNA-seq of 13 broiler testicular samples, including nFeature_RNA, nCount_RNA and percent.mt. B Information summary of 13 broiler testicular samples sequenced in this study. C A UMAP plot showing the annotated testicular cell types. Each dot represents a single testicular cell and is colored based on the five different ages of broilers. D Thirteen UMAP plots showing the annotated testicular cell types of each broiler sample sequenced in this study with color based on the cell type. E Immunofluorescence staining for SOX9, ACTA2, VWF, CLEC3B, DDX4, TOP2A, TPPP2 (red) and DAPI (blue) in testes. Scale bars = 100 μm. Fig. S2. Marker expression and percentage of each cluster of five different ages. A Marker expression patterns of each cluster on UMAP plots. A gradient of blue and gray represents high or low marker expression levels. B Bar plot showing the percentage of each cluster of five groups (1 d, 3 weeks, 12 weeks, 18 weeks, and 24 weeks). Fig. S3. Dynamic transcriptional characteristics of germ cell development in chicken testes. A Bar plot showing the number of testicular germ cells and percentage of each cluster from five groups (1 d, 3 weeks, 12 weeks, 18 weeks, and 24 weeks). The dashed line represents spermatocytes, while the solid line represents spermatids. B Heatmap showing the markers of each cell cluster of germ cells by “DoHeatmap” function. All markers are calculated by “FindAllMarker” function. The number on the left shows the number of differentially expressed genes and is colored based on its cell type. C UMAP plots showing the annotated cell types of spermatocytes and spermatids. Dashed lines and arrows represent their developmental trajectory. D Heatmaps showing the representative markers of each cell cluster of spermatocytes and spermatids. A gradient of red and blue represents high or low marker expression levels. E Expression patterns of representative dynami [file 40104_2025_1304_MOESM1_ESM.zip › Figure S4.tif]

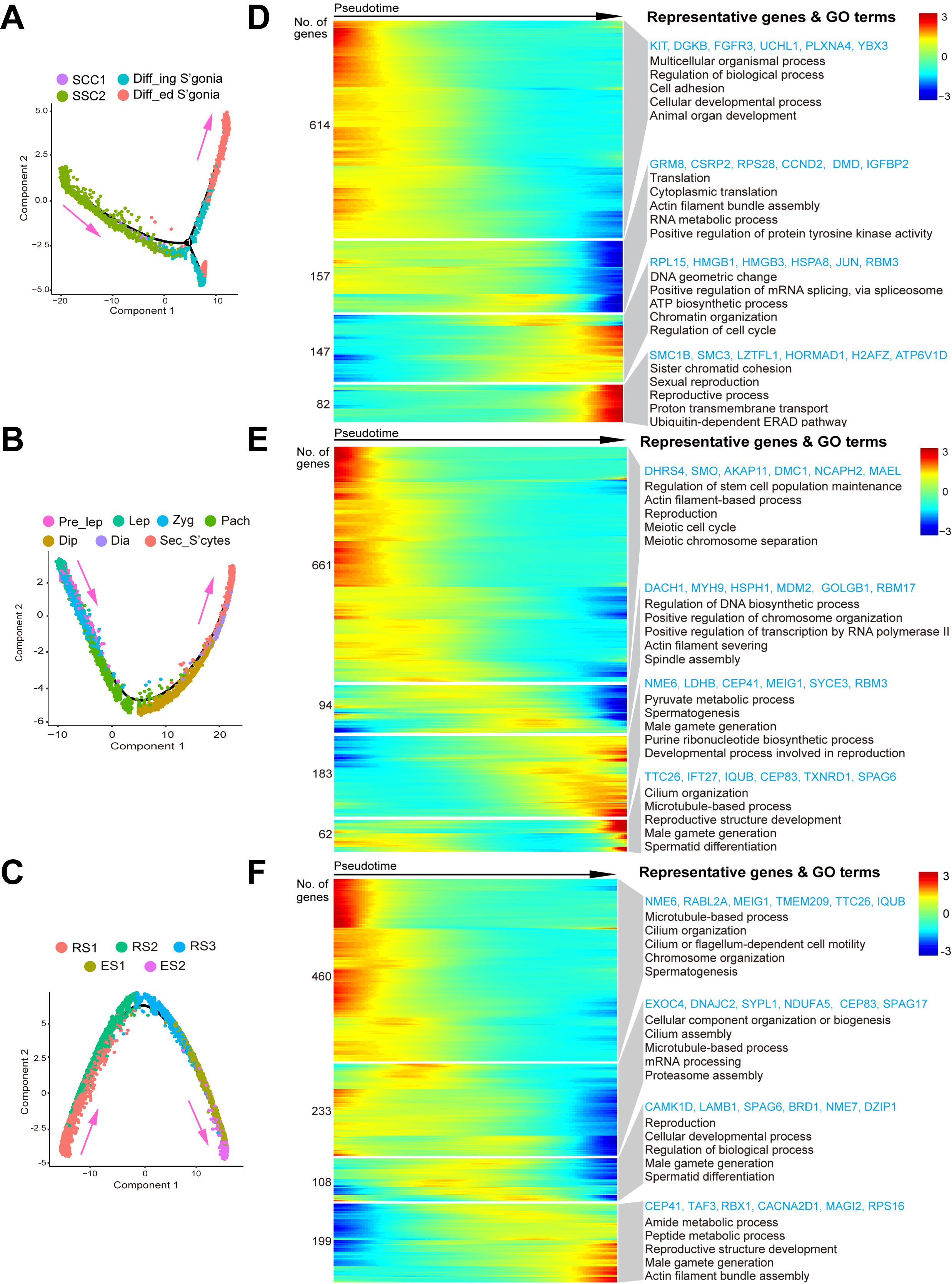

Supplement: Supplementary file 1 — Additional file 1: Fig. S1. Quality control and information of scRNA-seq. A Quality of scRNA-seq of 13 broiler testicular samples, including nFeature_RNA, nCount_RNA and percent.mt. B Information summary of 13 broiler testicular samples sequenced in this study. C A UMAP plot showing the annotated testicular cell types. Each dot represents a single testicular cell and is colored based on the five different ages of broilers. D Thirteen UMAP plots showing the annotated testicular cell types of each broiler sample sequenced in this study with color based on the cell type. E Immunofluorescence staining for SOX9, ACTA2, VWF, CLEC3B, DDX4, TOP2A, TPPP2 (red) and DAPI (blue) in testes. Scale bars = 100 μm. Fig. S2. Marker expression and percentage of each cluster of five different ages. A Marker expression patterns of each cluster on UMAP plots. A gradient of blue and gray represents high or low marker expression levels. B Bar plot showing the percentage of each cluster of five groups (1 d, 3 weeks, 12 weeks, 18 weeks, and 24 weeks). Fig. S3. Dynamic transcriptional characteristics of germ cell development in chicken testes. A Bar plot showing the number of testicular germ cells and percentage of each cluster from five groups (1 d, 3 weeks, 12 weeks, 18 weeks, and 24 weeks). The dashed line represents spermatocytes, while the solid line represents spermatids. B Heatmap showing the markers of each cell cluster of germ cells by “DoHeatmap” function. All markers are calculated by “FindAllMarker” function. The number on the left shows the number of differentially expressed genes and is colored based on its cell type. C UMAP plots showing the annotated cell types of spermatocytes and spermatids. Dashed lines and arrows represent their developmental trajectory. D Heatmaps showing the representative markers of each cell cluster of spermatocytes and spermatids. A gradient of red and blue represents high or low marker expression levels. E Expression patterns of representative dynami [file 40104_2025_1304_MOESM1_ESM.zip › Figure S5.tif]

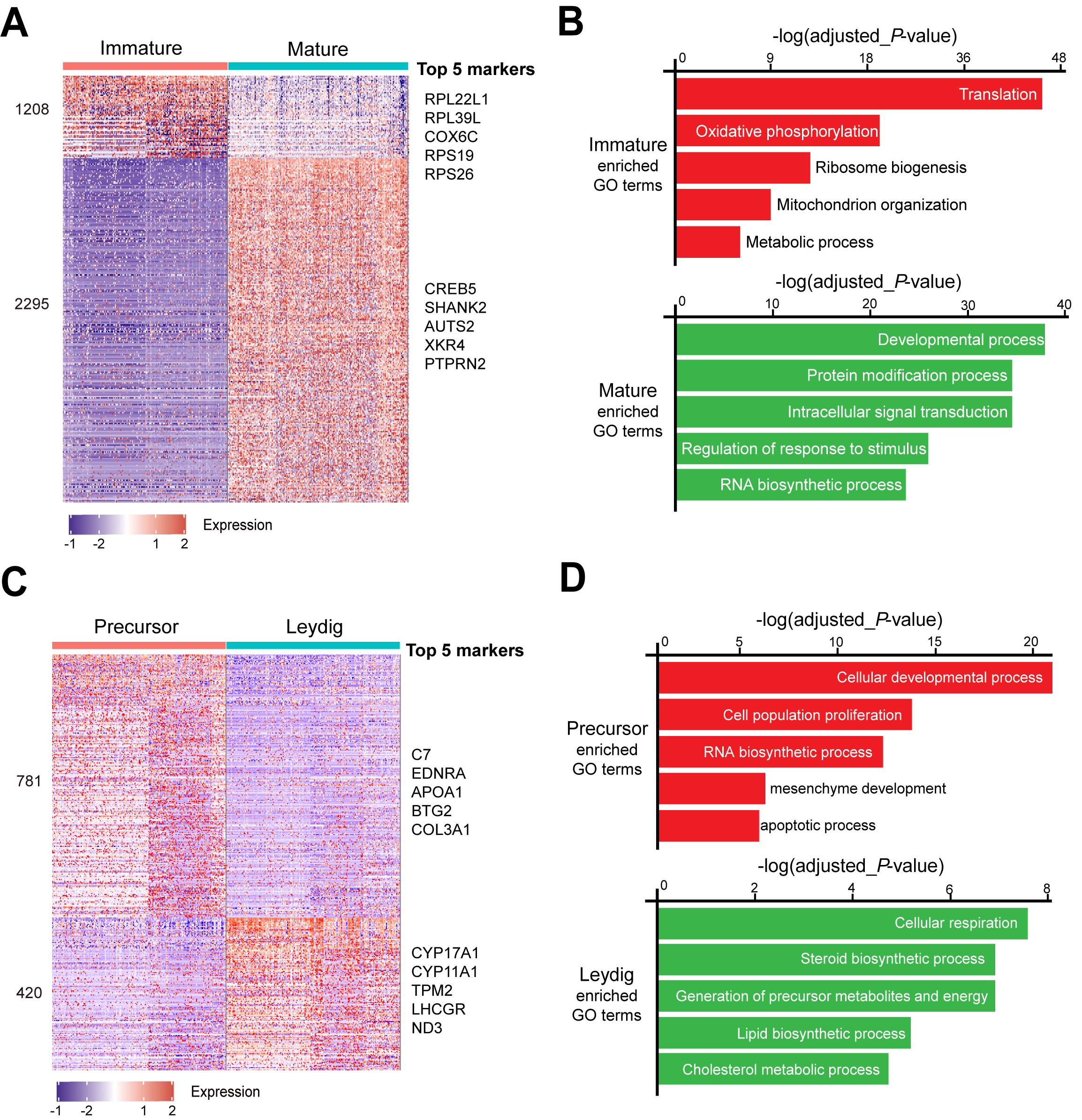

Supplement: Supplementary file 1 — Additional file 1: Fig. S1. Quality control and information of scRNA-seq. A Quality of scRNA-seq of 13 broiler testicular samples, including nFeature_RNA, nCount_RNA and percent.mt. B Information summary of 13 broiler testicular samples sequenced in this study. C A UMAP plot showing the annotated testicular cell types. Each dot represents a single testicular cell and is colored based on the five different ages of broilers. D Thirteen UMAP plots showing the annotated testicular cell types of each broiler sample sequenced in this study with color based on the cell type. E Immunofluorescence staining for SOX9, ACTA2, VWF, CLEC3B, DDX4, TOP2A, TPPP2 (red) and DAPI (blue) in testes. Scale bars = 100 μm. Fig. S2. Marker expression and percentage of each cluster of five different ages. A Marker expression patterns of each cluster on UMAP plots. A gradient of blue and gray represents high or low marker expression levels. B Bar plot showing the percentage of each cluster of five groups (1 d, 3 weeks, 12 weeks, 18 weeks, and 24 weeks). Fig. S3. Dynamic transcriptional characteristics of germ cell development in chicken testes. A Bar plot showing the number of testicular germ cells and percentage of each cluster from five groups (1 d, 3 weeks, 12 weeks, 18 weeks, and 24 weeks). The dashed line represents spermatocytes, while the solid line represents spermatids. B Heatmap showing the markers of each cell cluster of germ cells by “DoHeatmap” function. All markers are calculated by “FindAllMarker” function. The number on the left shows the number of differentially expressed genes and is colored based on its cell type. C UMAP plots showing the annotated cell types of spermatocytes and spermatids. Dashed lines and arrows represent their developmental trajectory. D Heatmaps showing the representative markers of each cell cluster of spermatocytes and spermatids. A gradient of red and blue represents high or low marker expression levels. E Expression patterns of representative dynami [file 40104_2025_1304_MOESM1_ESM.zip › Figure S6.tif]
